# Concurrent tunable structural color, luminescence, and afterglow in single ZnS: X@SiO2 spheres via SiO_2_‐nanoarmor and calcination decoupling collaborative strategy

**DOI:** 10.1002/smo2.70065

**Published:** 2026-06-24

**Authors:** Tianyi Liu, Rou Meng, Shufen Zhang, Suli Wu

**Affiliations:** ^1^ State Key Laboratory of Fine Chemicals Frontier Science Center for Smart Materials Dalian University of Technology Dalian China

**Keywords:** afterglow, doping engineering, luminescence, structural color, wurtzite ZnS: X@SiO_2_spheres

## Abstract

The integration of structural color, photoluminescence, and afterglow into a single material remains a fundamental challenge due to the conflicting requirements for structural integrity and high‐temperature crystallization. Here, we present a SiO_*2*_‐nanoarmor and calcination decoupling collaborative strategy to create monodisperse wurtzite‐type ZnS: X@SiO_2_ spheres (X = Ag^+^, Cu^2+^, Mn^2+^) as a unified, single‐particle optical platform. Spatially, a conformal SiO_2_ nanocoating acts as a thermally stable scaffold, preserving perfect spherical morphology during solid‐phase transformation at 1000°C, thereby enabling angle‐independent structural colors across the visible spectrum via Mie resonance. Calcination decoupling was achieved through an alternating O_2_/N_2_ atmosphere, which removes carbon deposits at low temperature and subsequently drives a complete sphalerite‐to‐wurtzite phase transition with 91% conversion, while preventing oxidation. Programmable doping introduces engineered energy levels, enabling tunable photoluminescence from 490 nm (blue) to 600 nm (orange) and white emission through co‐doping. The fluorescence quantum yield was 12.8%. Notably, Cu‐ and Mn‐doped spheres exhibit green afterglow lasting up to 12.27 s, and their duration was controlled by the content of the wurtzite phase. This intrinsic multifunctionality allows sophisticated multi‐channel optical encryption, where a single pattern sequentially displays distinct information under natural light, UV illumination, and afterglow conditions.

## Graphical abstract

For the first time, tunable structural color, luminescence, and afterglow are integrated into single ZnS:X@SiO_2_ spheres via a SiO_2_‐nanoarmor and calcination decoupling strategy, achieving 250–410 nm monodisperse wurtzite spheres with 91% phase conversion, 12.8% PLQY, and 12.27 s afterglow.

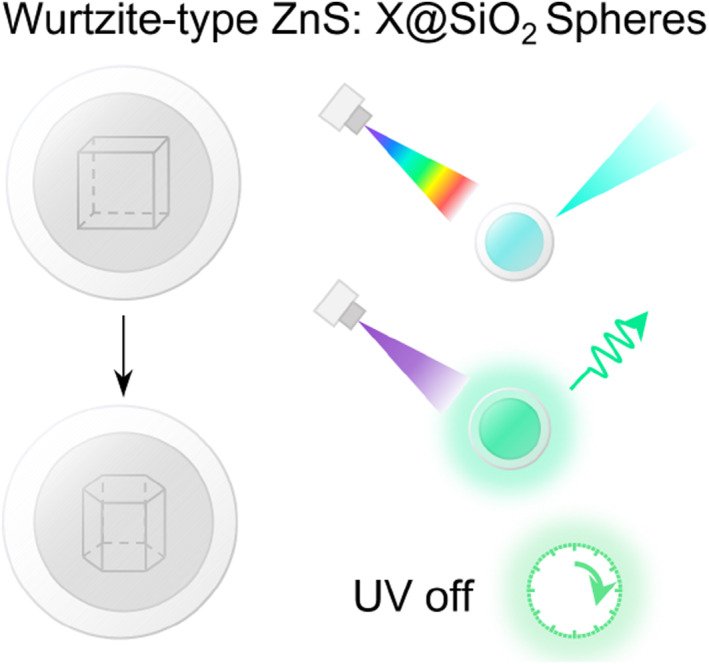

## 1 INTRODUCTION

Since approximately 540 million years ago, when living organisms attained the ability to perceive light, diverse light signals have played an essential role in their life processes, such as structural color and luminescence.[^1^, ^2^, ^3^] To this day, organisms have developed the ability to regulate a diverse range of light signals.[^4^, ^5^, ^6^, ^7^] Owing to the advantages of structural coloration and luminescence in biological activities, a limited number of organisms have acquired both of these capabilities, such as *Ctenophores*.[^8^, ^9^] The spines of *Ctenophores* display the beautiful iridescent structural color under natural light and emit a pale blue or pale green light at night. These phenomena were respectively attributed to the grating structure and jellyfish proteins. This captivating biological phenomenon has significantly increased the interest in integrating multiple light signals (such as structural colors, luminescence, and afterglow) within the same structure.[^10^, ^11^, ^12^, ^13^]

A prevalent strategy involves the incorporation of luminescent substances into colloidal photonic crystals, because the colloidal photonic crystals possess the merits of easily accessible raw materials for synthesis, stable structural colors, and a relatively straightforward self‐assembly process. On the one hand, fluorescent molecules can be selected to engage in the self‐assembly process of colloidal microspheres for the preparation of photonic crystals with luminescent properties.[^14^, ^15^] On the other hand, the fluorescent molecules can be pre—synthesized into colloidal microspheres prior to self‐assembly.[^16^, ^17^] Further, the elaborately designed multi—layered core—shell structure can even respond to multiple wavelengths of excitation light.^[18]^ For non‐molecular luminescent substances (e.g., lanthanide nanocrystals), external forces can induce their co‐assembly with colloidal microspheres, thus leading to the formation of ordered structures.[^19^, ^20^] Currently, the extensive research on the integration of structural colors and luminescent materials has yielded substantial progress in the multi‐stimulus response and independent control of structural colors and fluorescence, effectively enhancing the degree of information security.[^21^, ^22^] As the complexity of the composite structure increases, the manufacturing difficulty increases and the usage restrictions become more stringent. To overcome these inherent limitations associated with complex composites, it is essential to search for a single material that is inherently structured with structural color, luminescence, and afterglow.

To achieve this vision, ZnS could be the most suitable potential solution. On the one hand, due to the relatively high refractive index (∼2.37), it has been reported that sphalerite‐type ZnS (low‐temperature phase) spheres synthesized in the aqueous phase were utilized for generating Mie scattering structural colors and photonic crystal structural colors.[^23^, ^24^] Structural colors from Mie scattering took place when the scale of a substance was comparable to the wavelength of visible light. It was dependent on the coloration of individual particles rather than periodic repetitive structures, such as photonic crystals.^[25]^ On the other hand, as a widely used and reported luminescent material, the wurtzite‐type ZnS crystal (high‐temperature phase) can be doped with various ions (Ag^+^, Al^3+^, Cu^2+^, and Mn^2+^) to attain tunable luminescence and afterglow with a broad spectral response.[^26^, ^27^, ^28^, ^29^] Although ZnS has fulfilled the aforementioned requirements, the integration of structural color with luminescence/afterglow within a single material system has long encountered a fundamental challenge. Specifically, the morphological integrity necessary for generating vivid structural color and the high‐temperature crystallization process essential for achieving efficient luminescence are inherently contradictory in terms of thermodynamic driving forces and kinetic pathways.

**FIGURE 1:**
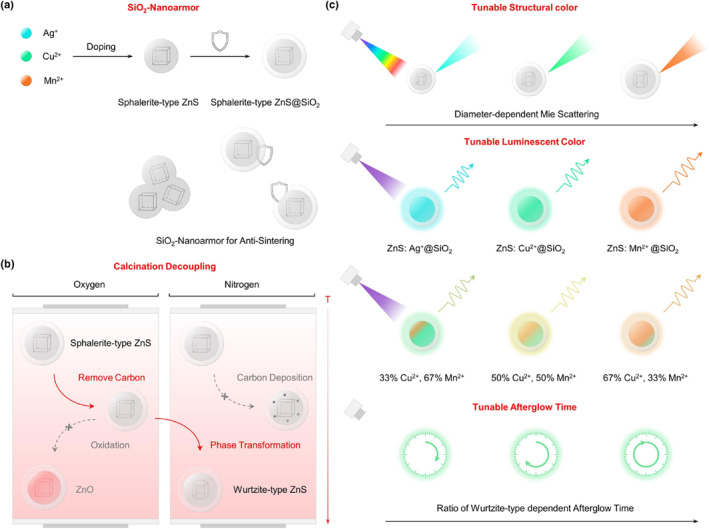
The design of Wurtzite‐type ZnS: X@SiO_2_ spheres with tunable structural color, luminescence color, and afterglow time. (a, b) Schematic diagram of our strategies for integrating structural colors with luminescence. Through the phase transformation process protected by the SiO_2_ shell and calcination decoupling, wurtzite‐type ZnS: X@SiO_2_ (X = Ag^+^, Cu^2+^, and Mn^2+^) spheres were fabricated to obtain only one material with the capabilities of tunable structural color, luminescence, and afterglow. (c) Schematic diagram depicting the general optical properties of wurtzite‐type ZnS: X@SiO_2_ (X = Ag^+^, Cu^2+^, and Mn^2+^) spheres. The structural color that covers the entire visible spectrum can be obtained by systematically adjusting the diameter of the spheres. The luminescent color can be precisely adjusted by doping with a single component or multiple components. The time of the afterglow was determined by the proportion of wurtzite‐type ZnS crystals.

Hence, through the SiO_2_‐nanoarmor and calcination decoupling collaborative strategy, we have achieved the integration of structural color, luminescence, and afterglow for the first time at the sub‐micron scale. Specifically, doping engineering was employed to introduce ions for the synthesis of sphalerite‐type ZnS: X (X = Ag^+^, Cu^2+^, and Mn^2+^) spheres during the aqueous‐phase synthesis. Subsequently, a SiO_2_ shell was applied as a nanoarmor to separate the ZnS spheres, which significantly improved their anti‐sintering capacity during the phase transition (ranging from 700 to 1000°C), thus preserving the spherical morphology (Figure 1a). The synthesized wurtzite‐type ZnS: X@SiO_2_ spheres were capable of attaining a diameter deviation of less than 10% within the range of 250–410 nm, which allows their Mie scattering structural color to be adjusted from 420 to 630 nm at the single‐sphere level. Furthermore, through the selective introduction of oxygen and nitrogen, the carbon deposition process and the high—temperature phase transformation process can be separated, thereby achieving calcination decoupling (Figure 1b). Here, decoupling calcination was defined as the separation of carbon removal in an oxygen atmosphere at a temperature below 600°C and the phase transformation of sphalerite to wurtzite in a nitrogen atmosphere at a temperature above 700°C. This strategy has led to an increase in the brightness of the sample powder from 0.22 to 1, with the oxidation rate of the ZnS crystal being close to 0. Through systematic introduction of single‐component or multi‐component ions, wurtzite‐type ZnS: X@SiO_2_ spheres demonstrate tunable luminescence ranging from 490 to 600 nm under UV light (PLQY = 12.8%). Wurtzite‐type ZnS: Cu and ZnS: Mn crystals demonstrate a green afterglow, and the duration of the afterglow can be determined by the proportion of wurtzite‐type ZnS crystals. The afterglow of the powder endures for a maximum of 12.27 s (Figure 1c). Overall, the wurtzite‐type ZnS: X@SiO_2_ sphere has achieved tunable structural color, luminescence, and afterglow at the sub ‐ micron scale for the first time. This compatibility with most processes paves the way for the design of intrinsic multifunctional optical materials.

## 2 RESULTS AND DISCUSSION

### 2.1 Synthesis of morphology‐stable ZnS: X@SiO_2_ spheres at high temperature

**FIGURE 2:**
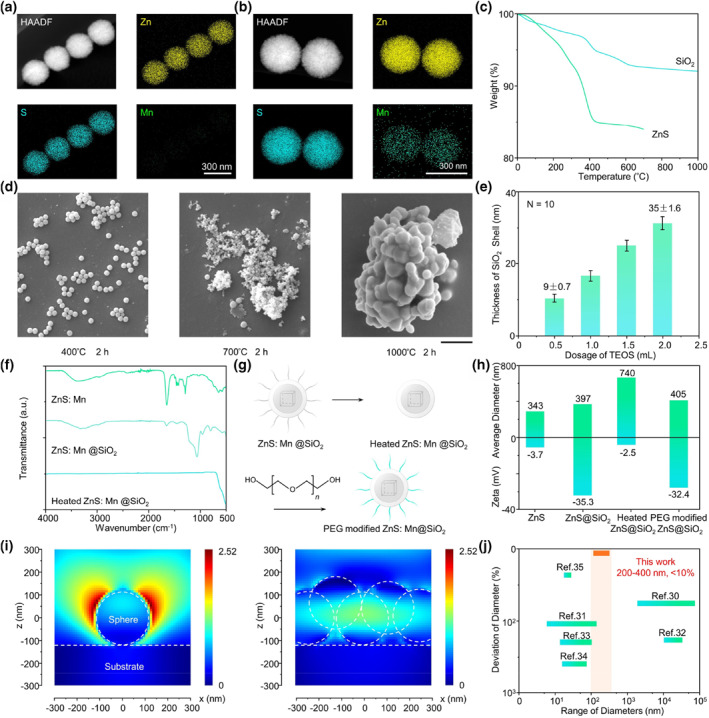
Controllable Synthesis of Morphology‐Stable ZnS: X@SiO_2_ Spheres at High Temperature. (a) transmission electron microscopy and the corresponding EDS Images of ZnS spheres. (b) TEM and the corresponding EDS Images of ZnS: Mn spheres. (c) Thermogravimetric curve of ZnS spheres and SiO_2_ spheres. Noted that further heating of ZnS will produce Zn vapor, which may cause damage to the machine. (d) SEM images of ZnS spheres under different temperature treatment. Noted that ZnS spheres undergo sintering and transform into amorphous crystals at 1000°C. Scale bar: 2 μm. (e) The relationship between the dosage of TEOS and the thickness of the SiO_2_ shell. The data was obtained from 10 samples. (f) FT‐IR spectra of ZnS: Mn, ZnS: Mn @SiO_2_, and heated ZnS: Mn@SiO_2_ spheres. (g) Schematic diagram of the ZnS: Mn@SiO_2_ sphere, heated ZnS: Mn@SiO_2_ sphere, and PEG modified ZnS: Mn@SiO_2_ sphere. (h) Average diameter and zeta potential of ZnS, ZnS@SiO_2_, Heated ZnS:@SiO_2_ and PEG modified ZnS@SiO_2_ spheres. (i) The simulated electromagnetic field distribution diagrams of a single ZnS sphere and multiple overlapping ZnS spheres. The overlapping state was determined by the amorphous ZnS crystals after sintering. (j) Comparison diagram of this work and the reported literature.

Our experiment commenced with the introduction of ions during the aqueous‐phase synthesis of ZnS, which was accomplished by adding the appropriate proportion of nitrates to the precursor solution. For instance, the EDS image of ZnS: Mn spheres directly indicates the uniform distribution of Mn elements, whereas no Mn element was detected in ZnS spheres (Figure 2a,b). An excessively elevated ion ratio can result in the loss of control over the spherical morphology of ZnS, which was ascribed to the uneven concentration of Zn^2+^ during the growth process of ZnS (Supporting Information S1: Figure S1). Note that the high‐resolution transmission electron microscopy (TEM) images indicate that the interplanar spacing of the 111 crystal plane measures 0.31 nm, which serves as direct evidence for the sphalerite ZnS crystals (Supporting Information S1: Figure S2). Therefore, calcination was necessary to facilitate the transformation from the spherical‐type ZnS to the wurtzite‐type ZnS crystal. However, at high temperatures, the ZnS spheres will decompose and release Zn vapor, whereas the SiO_2_ spheres exhibit almost no weight loss within the temperature range of 600–1000°C (Figure 2c). Therefore, it can be predicted that it was difficult for ZnS spheres to maintain their morphology during the calcination. The Ostwald ripening process leads to the coarsening of uncoated ZnS spheres at high temperature. The surfaces of smaller spheres exhibit a greater curvature, thereby possessing a higher chemical potential.^[30]^ This results in the release of Zn atoms and their subsequent re ‐ deposition onto the larger spheres. In contrast, the SiO_2_ shell layer can maintain its spherical shape owing to its excellent thermal stability. To verify this conjecture, SEM images of ZnS: Mn spheres that had been heated at different temperatures (400, 700, 1000°C) were selected (Figure 2d). As anticipated, the ZnS: Mn spheres experienced ligand decomposition upon being heated to 700°C. At this stage, their spherical morphology was disrupted. Subsequently, when heated further to 1000°C, the broken ZnS spheres re‐sintered and transformed into amorphous crystals. In contrast, a 35 nm SiO_2_ shell was applied to the ZnS: Mn spheres to improve the anti‐sintering performance (Supporting Information S1: Figure S3). The SiO_2_ shell suppresses Ostwald ripening through three mechanisms. First, it provided physical separation that prevents direct particle‐particle contact, eliminating the most efficient mass transport pathway. Second, the SiO_2_ layer act as a diffusion barrier, limiting the vapor‐phase transport of Zn between cores; the diffusion length of Zn in dense SiO_2_ was lower than in air by maintaining a uniform size distribution and isolating each core in its own microcavity, the shell reduces the chemical potential gradient that drives ripening—smaller ZnS cores inside the same or adjacent cavities cannot easily transfer Zn ions to larger cores across the stable SiO_2_ barrier. Noted that an excessively thin SiO_2_ shell was unable to fully prevent the sintering of ZnS: Mn spheres, which could be attributed to the interfacial stress during the phase transformation. Experimental results indicate that a SiO_2_ shell with a thickness of at least 30 nm was necessary to stabilize the morphology of the ZnS: Mn spheres at 1000°C (Supporting Information S1: Figure S4). The thickness of the SiO_2_ shell can be precisely adjusted by the quantity of the silicon source (Tetraethyl orthosilicate, TEOS) utilized (Figure 2e). Following the calcination process, no resonance peaks were detected in the FT‐IR spectrum, indicating that the ligands on the surface of the ZnS: Mn@SiO_2_ spheres have nearly completely vanished (Figure 2f). To enhance the dispersion of the ZnS: Mn@SiO_2_ spheres subsequent to calcination, the surfaces of the spheres were modified with polyethylene glycol (PEG) (Figure 2g). In the FT‐IR spectrum, the peaks at 3400 cm^−1^ and 952 cm^−1^ correspond to the vibration peaks of ‐OH groups and C–O bonds, respectively, which confirms the successful modification of the spheres with PEG (Supporting Information S1: Figure S5). The dispersibility and stability of the PEG‐modified ZnS: Mn@SiO_2_ spheres were both enhanced. The zeta potential decreased from −2.5 to −32.4 mV, and the hydrated average diameter decreased from 740 to 405 nm (Figure 2h). Overall, ZnS: Mn@SiO_2_ spheres featuring strong anti‐sintering ability and good dispersibility at high temperature have been successfully synthesized. The diameter of the synthesized spheres can be accurately regulated within the range of 250–410 nm, with a standard deviation of less than 10% (Supporting Information S1: Figure S6). Only spheres exhibiting such a high degree of morphological uniformity has the potential to generate structural colors.

To further illustrate the significance of morphology control, the electric field distribution and spectra of a single ZnS: Mn@SiO_2_ sphere and slightly sintered ZnS: Mn@SiO_2_ spheres were simulated via the Finite‐Difference Time‐Domain (FDTD) method (Figure 2i). The electric field distribution diagram demonstrates that the resonance intensity of the overlapped ZnS spheres after sintering was even lower than that of a single sphere. The simulated reflection spectrum also failed to exhibit distinct scattering peaks, which can be ascribed to the strong incoherent scattering resulted from the morphological destruction (Supporting Information S1: Figure S7). The scattering was caused by the Mie scattering of individual ZnS spheres, and this phenomenon can be directly observed using a dark‐field microscope (Supporting Information S1: Figure S8). Moreover, the directly observed scattered color was consistent with the simulated spectrum. In addition, by changing the diameter of ZnS spheres, the alteration in scattering color can be directly observed, which further verifies that the structural color stems from Mie scattering (Supporting Information S1: Figure S9). Furthermore, owing to the high refractive index of ZnS, the introduction of the SiO_2_ shell exerts nearly no influence on its electromagnetic resonance and structural color (Supporting Information S1: Figure S10 and S11). Based on the aforementioned results, the SiO_2_ shell effectively preserved the morphology of ZnS: Mn at high temperatures (1000°C) without exerting any influence on its optical properties. Therefore, in comparison with the existing methods for ZnS synthesis,[^31^, ^32^, ^33^, ^34^, ^35^, ^36^] this work has successfully reduced the standard deviation of its particle size by at least one order of magnitude (Figure 2j).

### 2.2 The Preparation of Wurtzite‐type ZnS: X@SiO_2_ spheres without carbon

**FIGURE 3:**
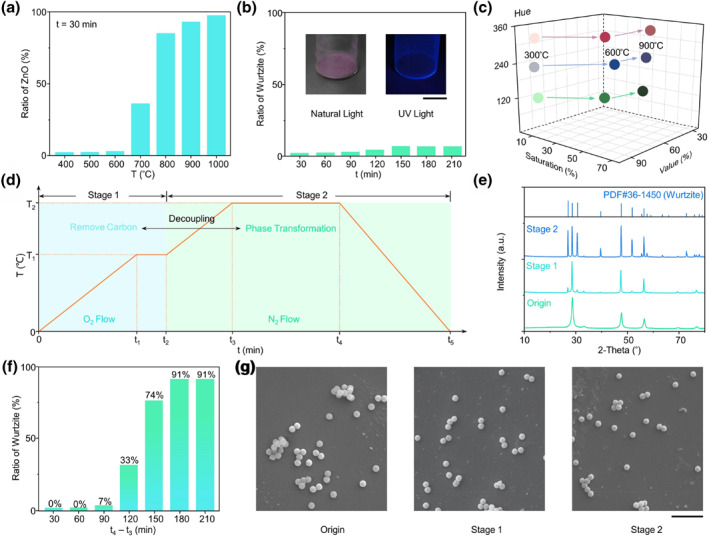
The Preparation of Wurtzite‐type ZnS: X@SiO_2_ Spheres without Carbon. (a) The ratio of ZnO in the sphalerite‐type ZnS: X@SiO_2_ spheres under the different calcination temperatures. (b) The ratio of wurtzite‐type ZnS in the sphalerite‐type ZnS: X@SiO_2_ spheres under the different calcination temperatures. Insets are the digital photographs of the sample under natural light and UV light. The carbon deposits have significantly impacted the structural color and luminescent properties of the sample. Scale bar: 1 cm. (c) HSV coordinates of samples with different calcination temperatures. (d) Schematic diagram of the calcination temperature of sphalerite‐type ZnS: X@SiO_2_ spheres with time in the oxygen and nitrogen atmosphere. (e) XRD spectra of ZnS: X@SiO_2_ spheres at different stages. (f) The ratio of wurtzite‐type ZnS in the sphalerite‐type ZnS: X@SiO_2_ spheres under the different calcination times. (g) SEM images of ZnS: X@SiO_2_ spheres at different stages. Scale bar: 2 μm.

It has been demonstrated that ZnS: Mn@SiO_2_ spheres exhibit remarkable anti‐sintering ability. However, in order to attain the luminescent performance, it is also essential to guarantee the removal of carbon deposits and prevent oxidation during the process of the crystal transformation from sphalerite‐type to wurtzite‐type ZnS. Initially, two preliminary experiments were carried out to explore the solutions. For the oxygen atmosphere, ZnS: Mn@SiO_2_ spheres undergo rapid oxidation to ZnO when calcined in an oxygen or air atmosphere. Owing to the protective effect of the SiO_2_ shell, ZnS did not exhibit any obvious oxidation even at 600°C. But at 1000°C, the ratio of conversion reached nearly 100% within 30 min (Figure 3a). Direct evidence was that the characteristic peaks of the XRD of the calcined sample corresponded to the standard card of ZnO (PDF#36‐1451) (Supporting Information S1: Figure S12). The pure oxygen environment led to the absence of carbon deposition in the prepared powder; however, its luminescence was extremely weak (Supporting Information S1: Figure S13). A prevalent strategy for preventing oxidation was the utilization of a nitrogen atmosphere. Although the products retained a spherical morphology (Supporting Information S1: Figure S14), heavy carbon deposition had a substantial impact on the structural color and luminescence performance of ZnS: Mn@SiO_2_, and hindered the transformation from sphalerite‐type to wurtzite‐type (Figure 3b). Carbon deposits significantly altered the absorption of the spheres, thereby causing a change in the color of the powders (Supporting Information S1: Figure S15). To precisely monitor the generation of sedimentary carbon, the saturation and brightness values of the prepared powder were assessed using the HSV (Hue, Saturation and Value) color space (Figure 3c). For instance, the value of the blue sample at 600°C was 0.49, while it was 0.41 at 900°C, which indicates that the majority of the carbon deposition was formed before 600°C.

To tackle the dilemma of oxidation and carbon deposition, the process decoupling strategy of selectively introducing oxygen and nitrogen was selected (Figure 3d). According to the results of the preliminary experiments, below 600°C, ZnS: Mn@SiO_2_ spheres were difficult to oxidize, and most of the carbon deposits were generated. Therefore, oxygen was introduced in Stage 1 to eliminate the majority of carbon deposits, whereas nitrogen was introduced at a higher temperature (Stage 2) to facilitate the ZnS crystal form transformation from sphalerite‐type to wurtzite‐type without oxidation. The XRD patterns confirmed this assumption (Figure 3e). After Stage 1, the powder of ZnS: Mn@SiO_2_ spheres retained its white color, and there was almost no phase transformation. In Stage 2, almost all the crystals were transformed into the wurtzite phase, inducing the sample to emit orange light (Supporting Information S1: Figure S16). The proportion of wurtzite can be accurately controlled by the calcination time, and the maximum conversion rate can reach 91% (Figure 3f and Supporting Information S1: Figure S17). The SEM images indicate that the synthesized wurtzite‐type ZnS: Mn@SiO_2_ spheres retained a well ‐ defined spherical morphology during the entire calcination process (Figure 3g). By altering the doping elements, the wurtzite‐type ZnS: Cu@SiO_2_ spheres and ZnS: Ag@SiO_2_ can be synthesized in the same manner. Note that the heating and cooling rates should be regulated at 10°C per minute. Excessive heating rate may lead to the breakage of the spheres (Supporting Information S1: Figure S18).

### 2.3 Luminescence and Afterglow Properties of Wurtzite‐type ZnS: X@SiO_2_ spheres

**FIGURE 4:**
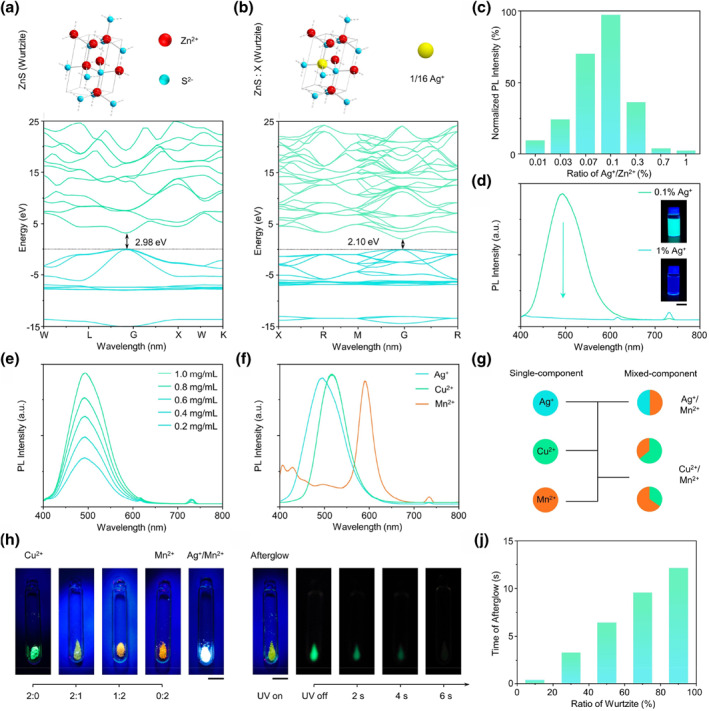
Luminescence and Afterglow Properties of Wurtzite‐type ZnS: X@SiO_2_ Spheres. (a) The crystal structure diagram of wurtzite‐type ZnS crystals (upper) and the simulated energy band diagram (lower). The red ball represents Zn^2+^ and the blue ball represents S^2−^. (b) The crystal structure diagram of wurtzite‐type ZnS: Ag^+^ crystals (upper) and the simulated energy band diagram (lower). The yellow ball represents Ag^+^. Noted that one of the 16 Zn^2+^ was replaced by Ag^+^ for the precise calculation. (c) Normalized PL intensity of wurtzite‐type ZnS: Ag^+^@SiO_2_ spheres with different ratio of Ag^+^/Zn^2+^(0.01%, 0.03%, 0.07%, 0.1%, 0.3%, 0.7%, and 1%). (d) PL spectra of wurtzite‐type ZnS: Ag^+^(0.1% and 1%)@SiO_2_ spheres. Insets are the digital photographs of the corresponding solution. Scale bar: 1 cm. (e) PL spectra of wurtzite‐type ZnS: Ag^+^(0.1%)@SiO_2_ spheres with different concentrations (0.2, 0.4, 0.6, 0.8, 1.0 mg/mL). (f) PL spectra of wurtzite‐type ZnS: Ag^+^@SiO_2_, ZnS: Cu^2+^@SiO_2_, and ZnS: Mn^2+^@SiO_2_ spheres. (g) Schematic diagram of single‐component doping and mixed‐component doping. (h) Digital photographs of wurtzite‐type ZnS: Cu^2+^, Mn^2+^@SiO_2_ and ZnS: Ag^+^, Mn^2+^@SiO_2_ spheres under UV light. Scale bar: 1 cm. (i) Digital photographs of wurtzite‐type ZnS: Cu^2+^, Mn^2+^@SiO_2_ under UV excitation and after turning off the UV light. Scale bar: 1 cm. (j) Relationship between the time of afterglow and the ratio of wurtzite‐type ZnS crystals.

Uniform wurtzite‐type ZnS: X@SiO_2_ (X = Ag^+^, Cu^2+^, Mn^2+^) spheres have been successfully fabricated. As the literature reports, diverse doping elements can induce new energy levels in the band gap of wurtzite‐type ZnS, thereby emitting different light under external stimulation. The simulated crystal band diagram visually demonstrates the introduction of new energy levels and the reduction of the wurtzite‐type ZnS band gap from 2.98 to 2.10 eV (Figure 4a,b). Noted that there exists a discrepancy between the simulated band gap width of wurtzite‐type ZnS crystals and the actual band gap width owing to the approximation process of density functional theory (DFT). Consequently, after applying the scissors difference operator for correction, the band gap width of wurtzite‐type ZnS: Ag was 2.82 eV (Supporting Information S1: Figure S19). The reduction in the band gap of wurtzite‐type ZnS can be ascribed to the formation of shallow traps by the acceptor energy levels introduced by Ag^+^. When UV light excites the material to generate free electron‐hole pairs, the holes are captured by the acceptor energy levels and engage in radiative recombination, emitting blue light at 490 nm (Supporting Information S1: Figure S20). While Cu^2+^ and Mn^2+^ can substitute for Zn^2+^, upon exposure to UV light, the energy was directly transferred to the d electrons of the dopant elements, leading to their transition to the excited state. Subsequently, photons were emitted through a radiative transition.[^28^, ^37^] Through systematically adjusting the proportion of the doping elements, the optimal doping amounts of Ag^+^, Cu^2+^, and Mn^2+^ were ascertained to be 0.1%, 0.1%, and 1%, respectively (Figure 4c and Supporting Information S1: Figure S21). An excessively elevated doping concentration will result in an augmentation of the probability of resonant energy transfer and non‐radiative transitions, giving rise to macroscopic manifestations like luminescence quenching. For example, ZnS: Ag@SiO_2_ spheres doped with 1% Ag^+^ scarcely emit any light (Figure 4d). Excessive Cu^2+^ and Mn^2+^ will lead to a similar phenomenon (Supporting Information S1: Figure S22). Due to the outstanding dispersibility of the synthesized wurtzite‐type ZnS: X@SiO_2_ spheres, the luminescence intensity of their solution demonstrates a favorable concentration‐dependent relationship (Figure 4e and Supporting Information S1: Figure S23). The fluorescence quantum yield was 12.8% (Supporting Information S1: Figure S24). By optimizing the proportion and dosage of the doping elements, the luminescent colors ranging from 490 to 600 nm were obtained. Among them, the wurtzite‐type ZnS: X@SiO_2_ doped with single components of Ag^+^, Cu^2+^, and Mn^2+,^ respectively, emit blue, green, and orange light (Figure 4f). Note that the weak luminescence within the blue range of wurtzite‐type ZnS: Mn^2+^@SiO_2_ is attributed to the presence of Zn^2+^ vacancies. To achieve a greater diversity of light colors, a mixed‐component doping method can be employed (Figure 4g). For instance, as the ratio of the doping amounts of Cu^2+^ and Mn^2+^ was adjusted from 2:0 to 2:1, 1:2, and 0:2, the corresponding emission colors of wurtzite‐type ZnS: Cu^2+^, Mn^2+^@SiO_2_ spheres were green, yellow‐green, yellow, and orange, respectively (Figure 4h).

The mixed‐component system composed of Ag^+^ and Mn^2+^ demonstrated white luminescence. Surprisingly, both wurtzite‐type ZnS: Cu^2+^@SiO_2_ spheres and wurtzite‐type ZnS: Mn^2+^@SiO_2_ spheres exhibit afterglow properties (Figure 4i). The energy of UV light was not only used to form the luminescent centers composed of the doped elements, but also generated holes and electrons, which were, respectively, captured by the trap energy levels and the luminescent centers. The maximum lifetime of the afterglow can reach 12.27 s (Supporting Information S1: Figure S25). Further, the lifetime of the afterglow can also be controlled by the proportion of wurtzite‐type ZnS (Figure 4j). Noted that the observed monotonic increase in afterglow lifetime with wurtzite content was not solely attributable to the phase fraction itself. As demonstrated by relatived literitures,[^38^, ^39^] heat‐induced sphalerite‐to‐wurtzite transformation in ZnS leads to a progressive increase in both the number and depth of trapping peaks from six peaks (0.98–1.45 eV) to eight peaks (0.95–1.56 eV) in deconvoluted thermoluminescence glow curves. These results directly establish that the phase transformation in ZnS induces a redistribution of trap states, not a simple averaging between two phases. Therefore, the progressive increase in afterglow time with wurtzite content likely reflected an evolution in trap distribution accompanying the phase transformation.

### 2.4 Diverse application of uniform wurtzite‐type ZnS: X@SiO_2_ spheres

It has been demonstrated that the wurtzite‐type ZnS: X@SiO_2_ spheres exhibit adjustable luminescence and afterglow properties, and the uniform spheres are capable of generating structural colors based on Mie scattering. The relationship between the resonant peak (*λ*) of Mie scattering and the diameter (*d*) of the sphere was presented by Formula (1).

(1) λ≈nd

where *n* represents the refractive index of the wurtzite‐type ZnS spheres, which was approximately 2.0. By calculation, wurtzite‐type ZnS: X@SiO_2_ spheres with diameters of 250, 270, 300, and 350 nm were synthesized, and their Mie scattering peaks corresponded to 420, 450, 530, and 630 nm, respectively (Supporting Information S1: Figure S26).

**FIGURE 5:**
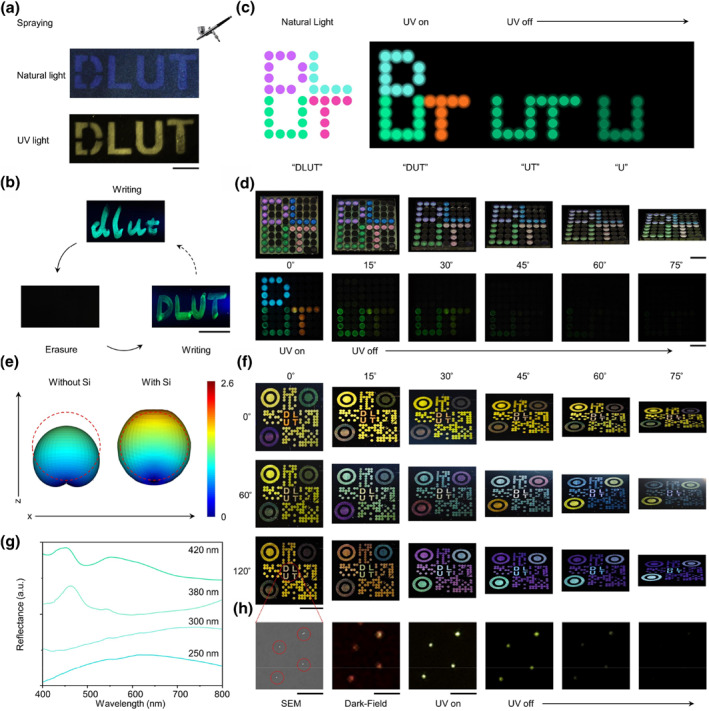
Diverse Application of Wurtzite‐type ZnS: X@SiO_2_ Spheres Integrating Tunable Structural Color, Luminescence, and Afterglow. (a) Digital Photographs of “DLUT” shaped pattern fabricated by spraying ZnS: Mn^2+^@SiO_2_ spheres. The pattern exhibits a purple hue under natural light and emits a yellow upon exposure to UV light. Scale bar: 1 cm. (b) Digital Photographs of the writing and erasure stages. Scale bar: 1 cm. (c) Schematic illustration of the multi‐channel information encryption by tunable structural color, luminescence, and afterglow. First, the pattern exhibits the information “DLUT” under natural light. Then, the pattern displays the information “DUT” under UV light. Finally, once the UV was off, it gradually transformed into “UT” and “U”. (d) Digital Photographs of the prepared pattern under natural light (upper), UV light, and UV off conditions (lower). The colors remain consistent when viewed from different angles under natural light due to the disordered structure. Scale bar: 1 cm. (e) Simulated far‐field scattering diagrams of ZnS spheres on vacuum and Si substrates. (f) Digital Photographs of multi‐colored patterns fabricated by ZnS spheres on Si substrates under different view angles (horizontal) and light source angle (vertical). Scale bar: 1 cm. (g) Reflectance spectra of ZnS spheres with different diameters. (h) SEM, Dark‐field microscope, and UV microscope image of 290 nm ZnS: Cu^2+^@SiO_2_ spheres.

At this stage, the well‐dispersed wurtzite‐type ZnS: X@SiO_2_ spheres have been successfully integrated with structural color, luminescence, and afterglow in the absence of any ordered structure, rendering them nearly compatible with any processing method. For instance, the sprayed “DLUT” pattern based on the wurtzite‐type 250 nm ZnS: Mn@SiO_2_ spheres can exhibit a blue color under natural light and a yellow color under UV light (Figure 5a). Further, the patterns written in can be erased and rewritten (Figure 5b). The combination of different types of optical signals can be employed to generate patterns containing multiple hidden messages (Supporting Information S1: Figure S27). The fabricated patterns were capable of sequentially displaying the multi‐color digital information of “DLUT”, “DUT”, “UT”, and “U” under natural light and UV light, and in the UV light‐off state, in accordance with the encoding (Figure 5c). It is worth noting that the angle‐dependence of the patterns under natural light was not significant, which can be ascribed to the disordered structure (Figure 5d). To further expand its response dimensions, Si substrates can be introduced to enhance the electromagnetic resonance of ZnS and alter its spatial distribution state, thereby achieving iridescent Mie scattering structural colors (Figure 5e). As depicted in Figure 5f, the structural colors of the fabricated multi‐color QR code pattern can vary individually with the angle of the light source and the viewing angle, displaying a diverse color appearance. The design of the QR code and the corresponding color spectra are presented in Supporting Information S1: Figures S28 and Figure 5g. The fabricated patterns are also capable of exhibiting scattering structural colors, luminescence, and afterglow at the single ‐ sphere level. The correspondence between the distribution of sphere positions in the SEM images and the distribution of scattering, luminescence, and afterglow positions provides direct evidence (Figure 5h). The microscopic random distribution and the macroscopic abundant colored appearance have significantly expanded the response dimensions and encryption level of optical anti‐counterfeiting labels.

## 3 CONCLUSION

In summary, we have established a novel materials design paradigm through the SiO_2_‐nanoarmor and calcination decoupling collaborative strategy, successfully integrating tunable structural color, programmable luminescence, and persistent afterglow within a single wurtzite‐type ZnS: X@SiO_2_ sphere. This approach fundamentally resolves the classic conflict between morphological preservation and high‐temperature activation. The SiO_2_ shell provides spatial isolation to maintain monodisperse spherical integrity during a 1000°C phase transition, enabling vivid Mie‐resonant structural colors, while the alternating O_2_/N_2_ atmosphere temporally separates carbon removal from crystal transformation, ensuring the wurtzite ZnS with excellent luminescent properties. Through rational doping engineering, we achieve a full palette of emission colors from blue to orange, including white light, and uncover tunable afterglow behavior linked to the host crystal phase. Crucially, this multifunctionality was encoded at the individual particle level, allowing each sphere to act as a standalone optical pixel. We demonstrate its powerful utility in advanced information encryption, where a single pattern can sequentially display different codes across multiple optical channels. The compatibility of this platform with diverse deposition methods and its ability to produce both angle‐independent and angle‐dependent structural colors further underscore its processing versatility and application potential. Beyond the specific ZnS system, this work presents a generalizable synthesis philosophy—decoupling conflicting processes—for designing next‐generation intrinsic multifunctional materials. It opens new avenues for creating compact, robust, and high‐performance optical units for future security technologies, dynamic displays, and integrated photonic devices.

## 4 EXPERIMENTAL SECTION

### 4.1 Controlled synthesis of ZnS MPs

ZnS MPs were generated by the reaction of thioacetamide (TAA) and Zn (NO_3_)_2_ in polyvinylpyrrolidone (PVP) solution. Additional experimental details are provided in the Supplementary Methods.

### 4.2 Characterization

The size, phase, and morphology of the ZnS spheres and the ZnS: X@SiO_2_ spheres (X = Ag^+^, Cu^2+^, Mn^2+^) were characterized by field emission scanning electron microscope (Nova Nanosem 450), TEM, selected‐area electron diffraction (SAED), and high‐resolution TEM (HR‐TEM, FEI TF30). The XRD patterns of the obtained ZnS spheres and the ZnS: X@SiO_2_ spheres (X = Ag^+^, Cu^2+^, Mn^2+^) were recorded using a Rigaku Smart Lab 9 kW diffractometer with Cu Kα radiation. Fourier‐transform infrared (FTIR) spectra were observed using a Fourier‐transform infrared spectrophotometer (FTIR‐6700, Nicolet). The zeta potential and the size of nanoparticles were analyzed by a Nanoparticle size and zeta potential analyzer (Nano‐ZS90). Darkfield microscope images were taken with a Nikon microscope Ni‐U. The reflection spectra of the samples were measured using a Hitachi U‐4100 spectrophotometer. Digital photos of the coatings were taken using a Smartphone (Mi 13).

## CONFLICT OF INTEREST STATEMENT

The authors declare no conflicts of interest.

## ETHICS STATEMENT

No animal or human experiments were involved in this study.

## Supporting information

Supporting Information S1

## Data Availability

The data that support the findings of this study are available from the corresponding author upon reasonable request.
