# Supplementary material for: Concurrent tunable structural color, luminescence, and afterglow in single ZnS: X@SiO2 spheres via SiO2‐nanoarmor and calcination decoupling collaborative strategy
Source: Smart Mol. 2026 Jun 24:e70065. Online ahead of print. doi: 10.1002/smo2.70065 (PMC13398616; doi:10.1002/smo2.70065)
Supplement: Supplementary file 1 — Supporting Information S1 [file SMO2-9999-0-s001.docx]

**Supporting Information**

**Concurrent Tunable Structural Color, Luminescence, and Afterglow in Single ZnS: X@SiO₂ spheres via Space-Process Dual Decoupling Strategy**

Tianyi Liu, Rou Meng, Shufen Zhang and Suli Wu^*^

**State Key Laboratory of Fine Chemicals, Frontiers Science Center for Smart Materials Oriented Chemical Engineering, Dalian University of Technology, Dalian 116024, China**

**Corresponding Author Email: wusuli@dlut.edu.cn**

**Contents**

I. Materials.......................................................................................................................................S1

II. Methods.......................................................................................................................................S1

III. Characterization.........................................................................................................................S6

IV. Supplementary Figures...............................................................................................................S7

**I. Materials**

**Chemicals.** Zn (NO_3_)_2_·6H_2_O, AgNO_3_, Cu (NO_3_)_2_, Mn (NO_3_)_2_ and nitric acid (≈68%) were purchased from Tianjin Damao Chemical Reagents. Thioacetamide (TAA), Ethylene glycol and n-butanol were purchased from Sinopharm Chemical Reagent Co., Ltd. Polyvinylpyrrolidone (PVP), (CH_3_COO)_2_Cd, NaBH_4_ and K_2_TeO_3_ was purchased from Sigma–Aldrich.

**II. Methods**

**Optical Simulation.** The simulated reflectance spectra were calculated using the commercial FDTD software (Lumerical simulations). The refractive indices of ZnS were set as 2.0. The refractive indices of SiO_2_ were set as 1.46. The optical parameters of the remaining substances are obtained from the software built-in package. In the case of couples of ZnS colloidal spheres, the perfectly matched layers were imposed in all of the directions as the boundary condition. In both cases, a plane wave (300–900 nm) was placed above the structure for a reflection spectral calculation.

**Density functional theory (DFT) Simulation.** Density functional theory calculations were performed using Gaussian 09W. Becke’s three-parameter hybrid exchange function, the Lee-Yang-Parr correlation function (B3LYP) was employed to investigate binding energies, and 6-31G (d,p) basis set was chosen. Dispersion-corrected methods DFT-D3 were employed in the calculation process. All the molecules were separately constructed, geometrically optimized, and energy calculation. An Independent gradient model based on the Hirshfeld partition of molecular density (IGMH) (Multiwfn methods and VMD visualization program) was used to identify weak non-covalent interactions.

**Synthesis of Sphalerite-type ZnS Colloidal Spheres.** First, 8.00 g of PVP was dissolved in 300 mL of deionized water at room temperature. After fully dissolved, the PVP solution was transferred to a three-necked flask and heated to 70℃ by a thermostatic magnetic stirrer. Next, 10.00 g of thioacetamide (TAA) was added into the flask and dissolved in the solution at 70℃. Then, after 30 minutes, 400 μL of nitric acid aqueous solution was injected into the flask. Finally, 20 mL of 2 mol/L Zn (NO_3_)_2_ solution was quickly poured into the flask under vigorous stirring conditions for 120 s. After 3h of reaction with gentle stirring, 20-40 mL of 2 mol/L Zn (NO_3_)_2_ solution was injected into the flask to get ZnS nanospheres of befitting diameter.

**Synthesis of** **Sphalerite-type ZnS:X (X = Ag^+^, Cu^2+^, Mn^2+^) Spheres.** First, 8.00 g of PVP was dissolved in 300 mL of deionized water at room temperature. After fully dissolved, the PVP solution was transferred to a three-necked flask and heated to 70℃ by a thermostatic magnetic stirrer. Next, 10.00 g of thioacetamide (TAA) was added into the flask and dissolved in the solution at 70℃. Then, after 30 minutes, 400 μL of nitric acid aqueous solution was injected into the flask. Finally, 20 mL of 2 mol/L Zn (NO_3_)_2_ solution was quickly poured into the flask under vigorous stirring conditions for 120 s. After 3h of reaction with gentle stirring, 20-40 mL of 2 mol/L Zn (NO_3_)_2_ and AgNO_3_/Cu (NO_3_)_2_/Mn (NO_3_)_2_ solution was injected into the flask to get ZnS nanospheres of befitting diameter.

**Synthesis of** **Sphalerite-type ZnS:X@SiO_2_ (X = Ag^+^, Cu^2+^, Mn^2+^) Spheres.** ZnS Colloid spheres (1.0 g) was dispersed in 200 mL of absolute ethanol. Then, 7.5 mL of ammonia water and 15 mL of deionized water were added into the dispersion. After 1 h of mixing, TEOS was added to the reaction system. Next, the reaction was carried out at 25 °C for 10 h. Finally, the ZnS:X@SiO_2_ (X = Ag^+^, Cu^2+^, Mn^2+^) colloidal spheres were collected through multiple centrifugations and washing as well as the final drying process.

**Crystal Phase Transformation of ZnS:X@SiO_2_ (X = Ag^+^, Cu^2+^, Mn^2+^) Colloidal Spheres.** The powder samples were placed in a tube furnace for calcination to promote the crystal phase transformation of ZnS, and then taken out after cooling.

**Preparation of the printing inks.** To begin, a mixture of deionized water (1 mL), n-butanal (2 mL), and ethylene glycol (2 mL) was prepared in a 10 mL reagent bottle. Sodium dodecyl sulfonate (0.02 g) was then added to the bottle, ensuring a homogeneous mixture under gentle stirring. Finally, MPs (0.05 g) were introduced and dispersed into the solution under ultrasonication, resulting in a uniform ink solution.

**Direct-ink-writing process.** The direct-ink-writing was conducted using the SIJ Technologies S050 model. Si wafers were meticulously cleaned with deionized water, ethanol, and acetone, followed by thorough drying, to ensure a pristine substrate surface. The printing was performed in bump mode, using a 75% square waveform at a frequency of 1 kHz, unless otherwise specified. The printer operated in a controlled clean room environment at room temperature with a relative humidity above 60%, minimizing the influence of environmental variables during printing.

**III. Characterization**

The size, phase, and morphology of the ZnS spheres and the ZnS:X@SiO_2_ spheres (X = Ag^+^, Cu^2+^, Mn^2+^) were characterized by field emission scanning electron microscope (Nova Nanosem 450), transmission electron microscopy (TEM), selected-area electron diffraction (SAED), and high-resolution TEM (HR-TEM, FEI TF30). The XRD pattern of the obtained ZnS spheres and the ZnS:X@SiO_2_ spheres (X = Ag^+^, Cu^2+^, Mn^2+^) were recorded using a Rigaku Smart Lab 9 kW diffractometer with Cu Kα radiation. Fourier-transform infrared (FTIR) spectra were observed using a Fourier-transform infrared spectrophotometer (FTIR-6700, Nicolet). The zeta potential and the size of nanoparticles were analyzed by a Nanoparticle size and zeta potential analyzer (Nano-ZS90). Darkfield microscope images were taken by Nikon microscope Ni-U. The reflection spectra of the samples were measured using a Hitachi U-4100 spectrophotometer. Digital photos of the coatings were taken using a Smartphone (Mi 13).

**IV. Supplementary Figures**


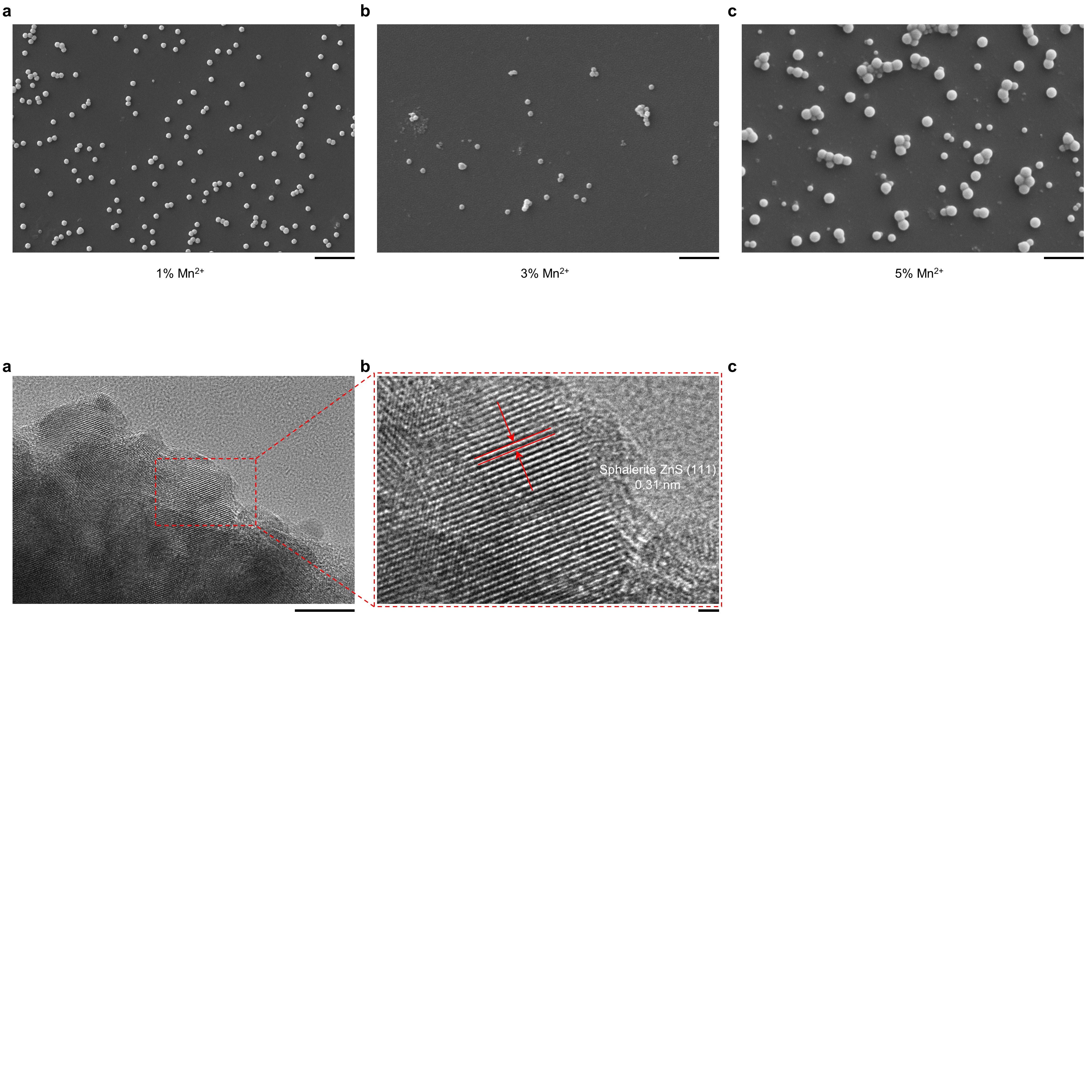


**Figure S1.** SEM images of ZnS:Mn spheres with different doping concentration. a) 1%, b) 3%, c) 5%. Scale bar: 2 μm.


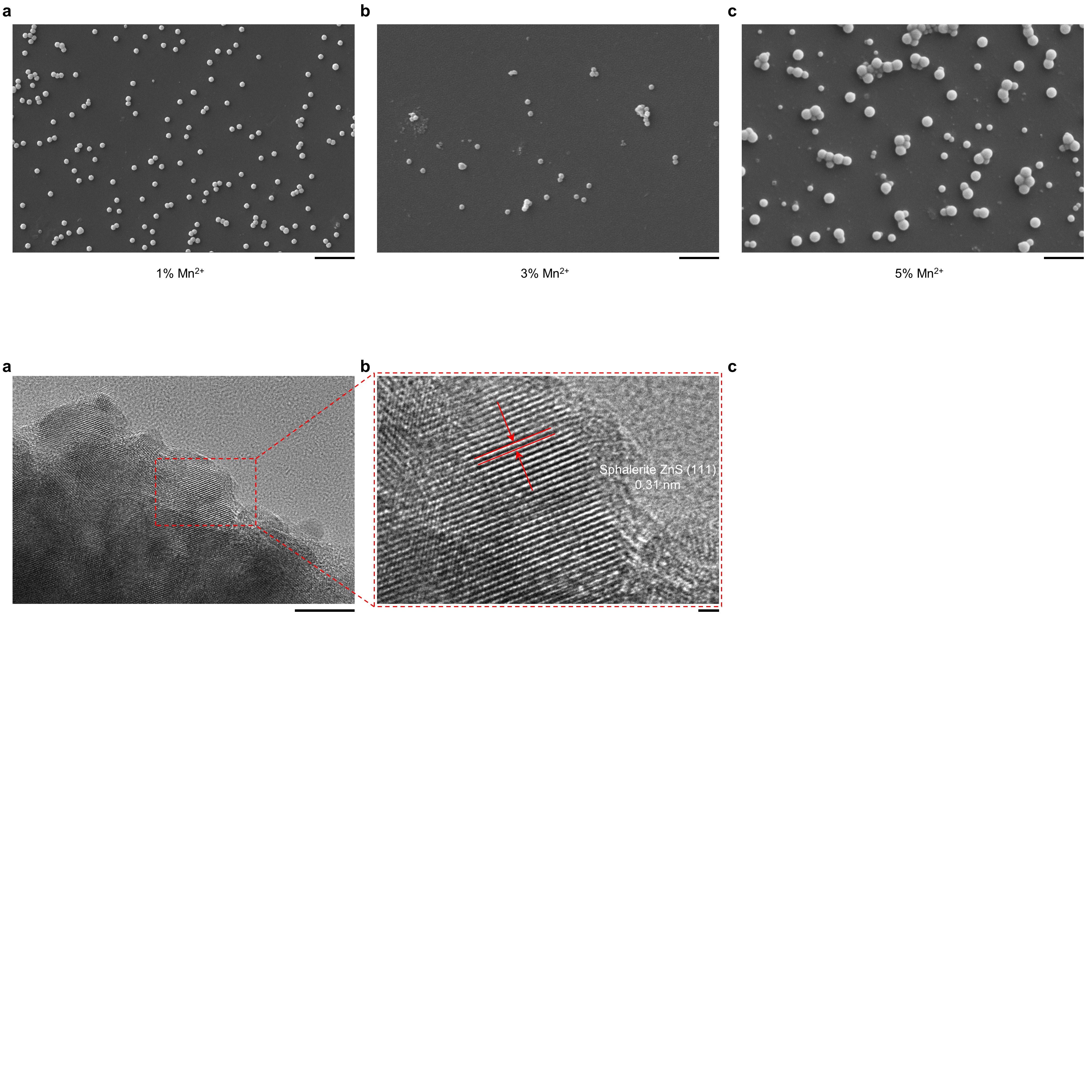


**Figure S2.** a-b) High-Resolution TEM images of sphalerite-type ZnS spheres. Scale bar: 10 nm, 1 nm.


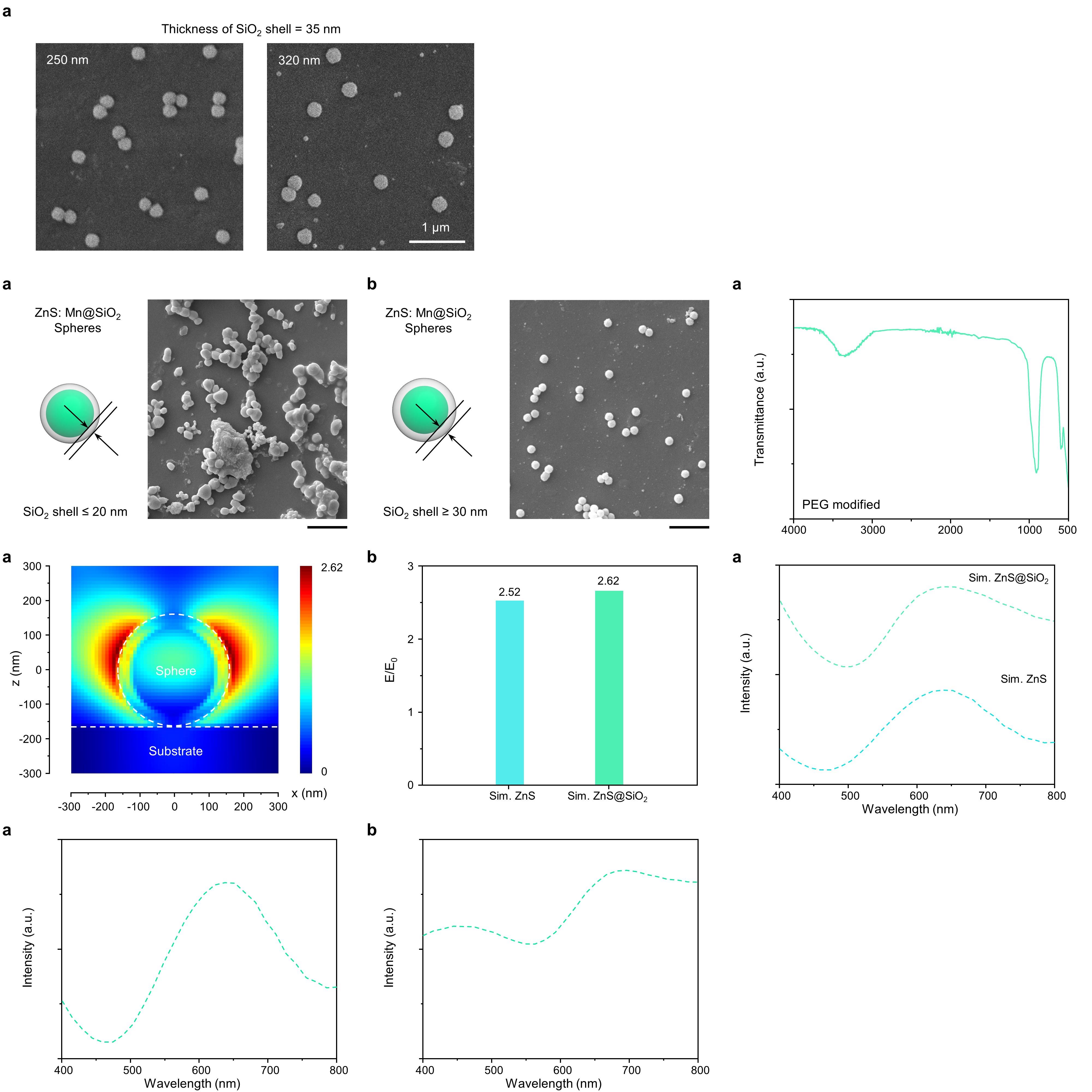


**Figure S3.** SEM images captured before and after the encapsulation of SiO_2_.


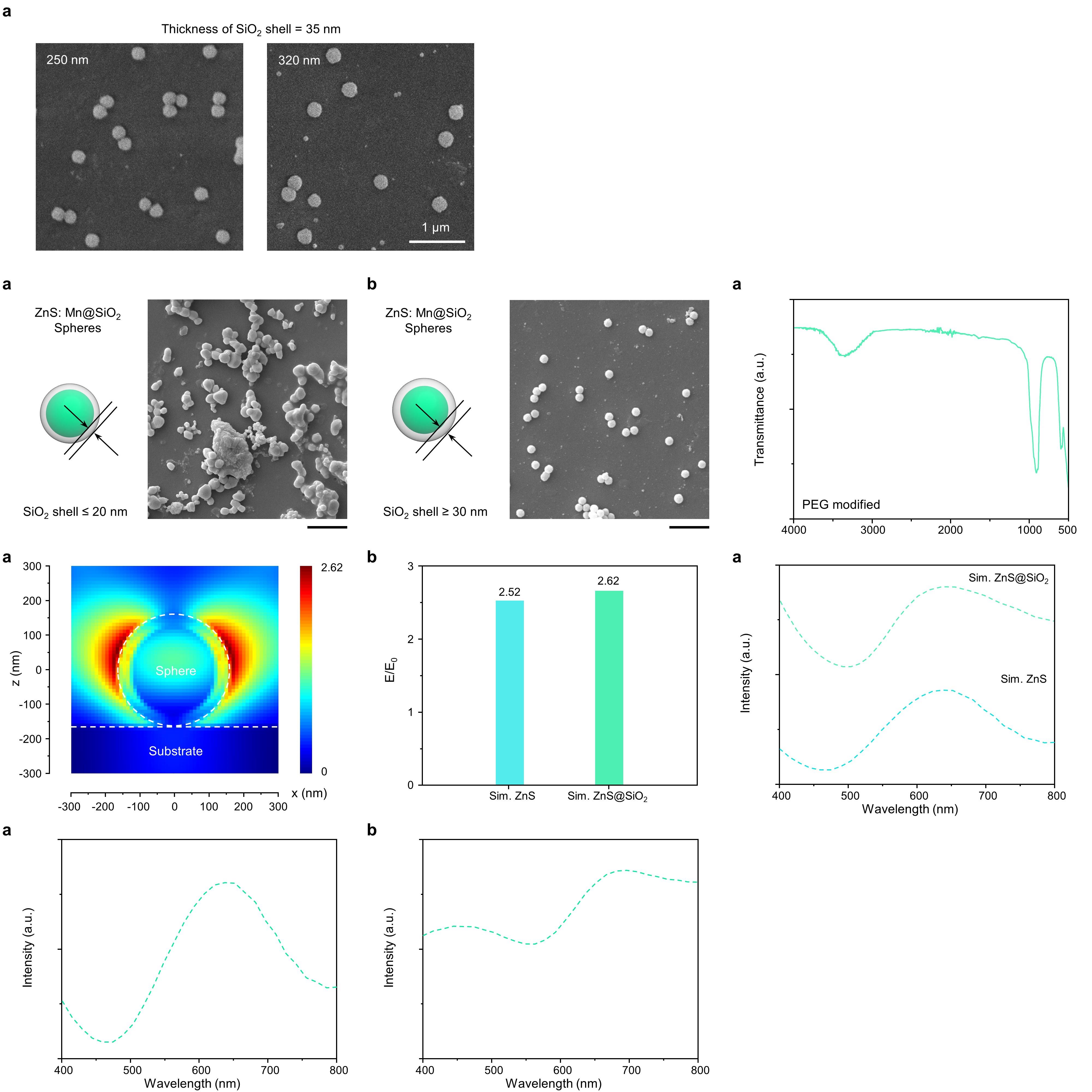


**Figure S4.** SEM images of ZnS: Mn@SiO_2_ spheres with different thicknesses of SiO_2_ shell after 1000 ̊C calcination. a) ≤20 nm, b) ≥30 nm. Scale bar: 1 μm.


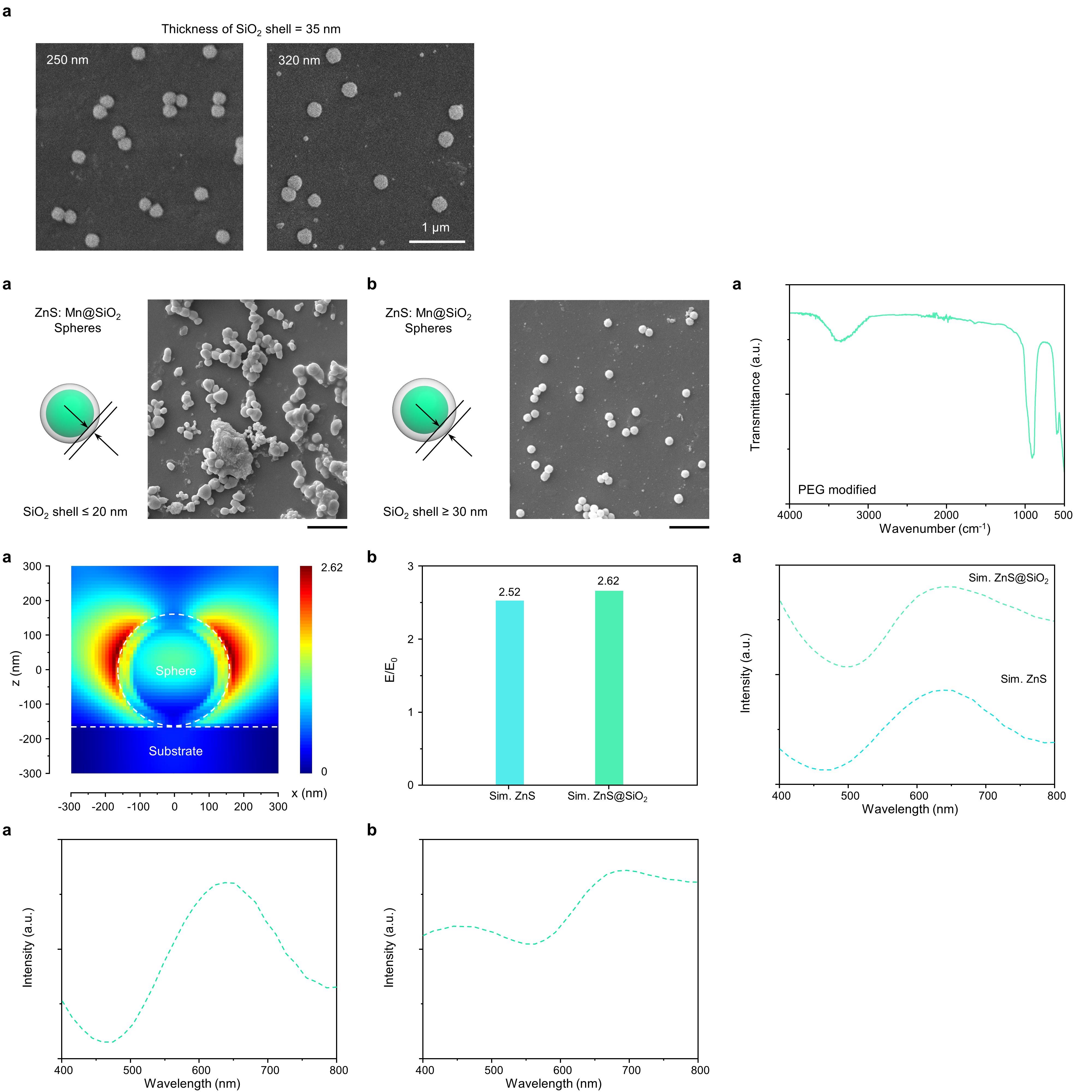


**Figure S5.** FT-IR spectrum of PEG-modified ZnS: Mn@SiO_2_ spheres.


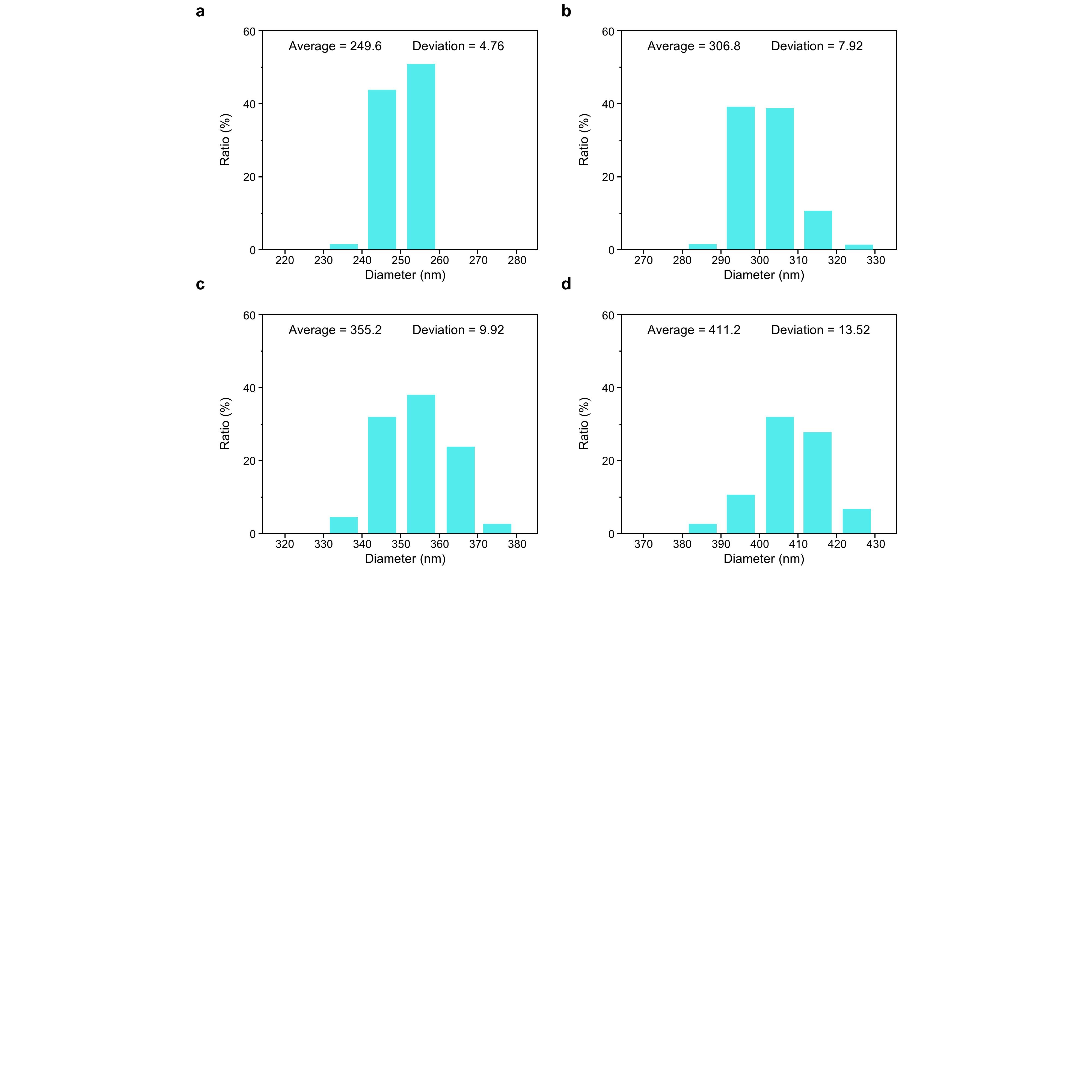


**Figure S6.** Diameter distribution of ZnS: Mn@SiO_2_ spheres with different diameter. a) 250 nm, b) 300 nm, c) 350 nmm d) 410 nm. Scale bar: 1 μm.


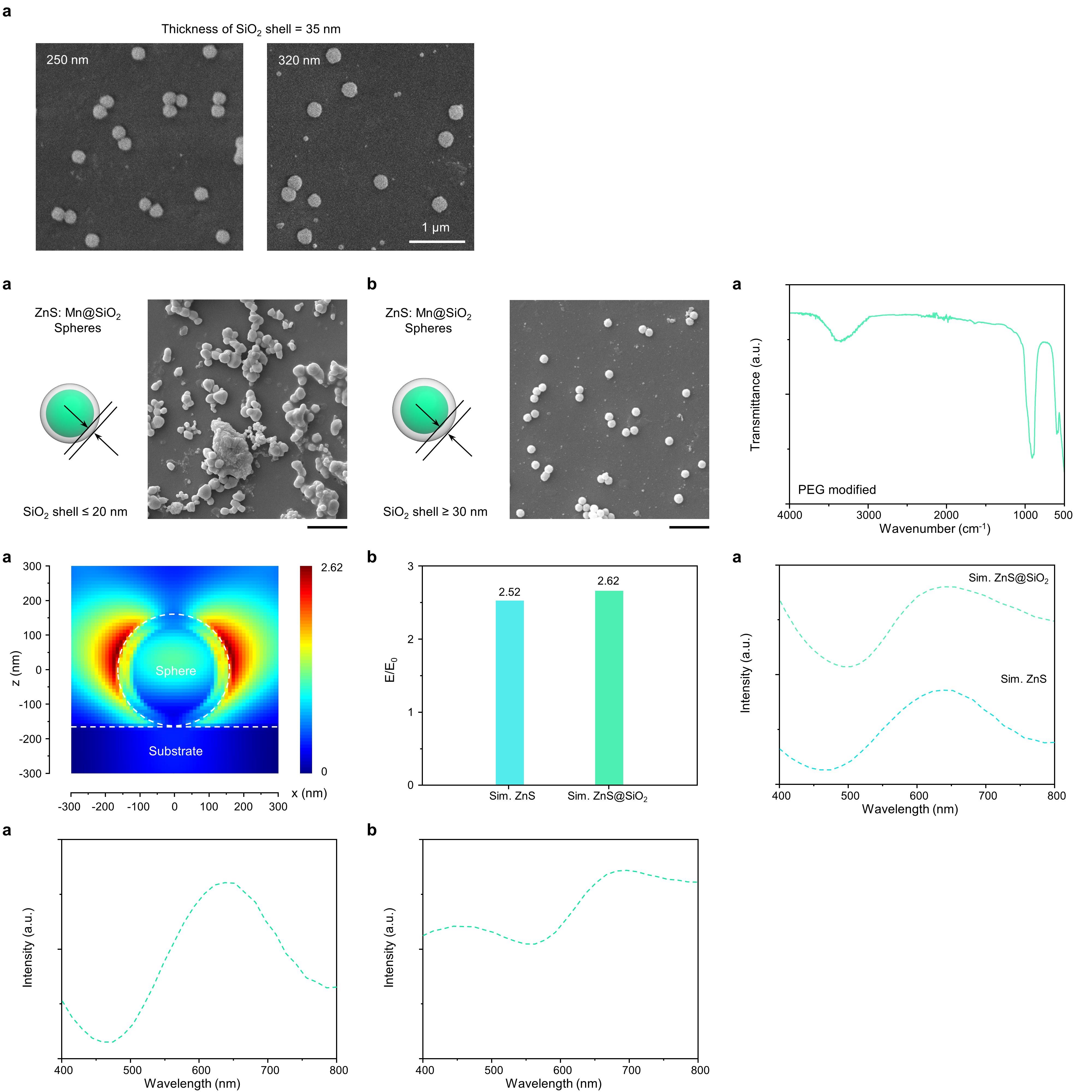


**Figure S7.** Simulated reflectance spectra of ZnS spheres. a) a single ZnS spheres, b) several overlapping ZnS spheres.


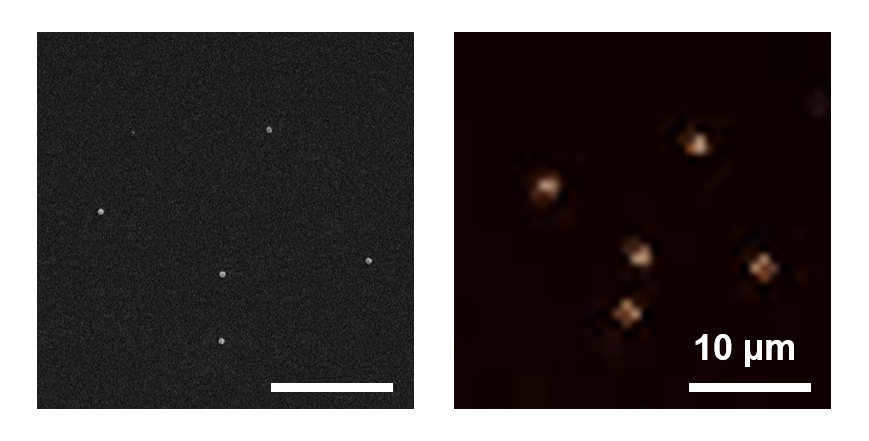


**Figure S8.** The SEM image and the corresponding dark-field scattering image of 250 nm ZnS spheres.


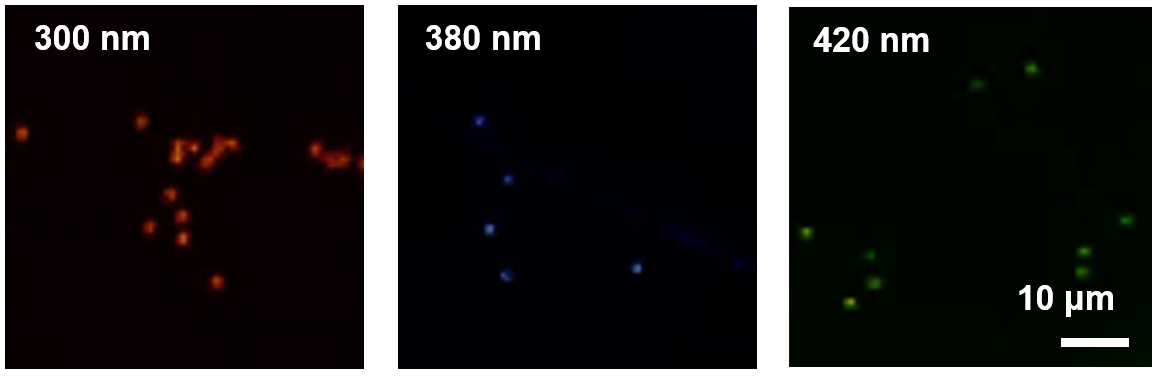


**Figure S9.** The dark-field scattering images of 300, 380, 420 nm ZnS spheres.


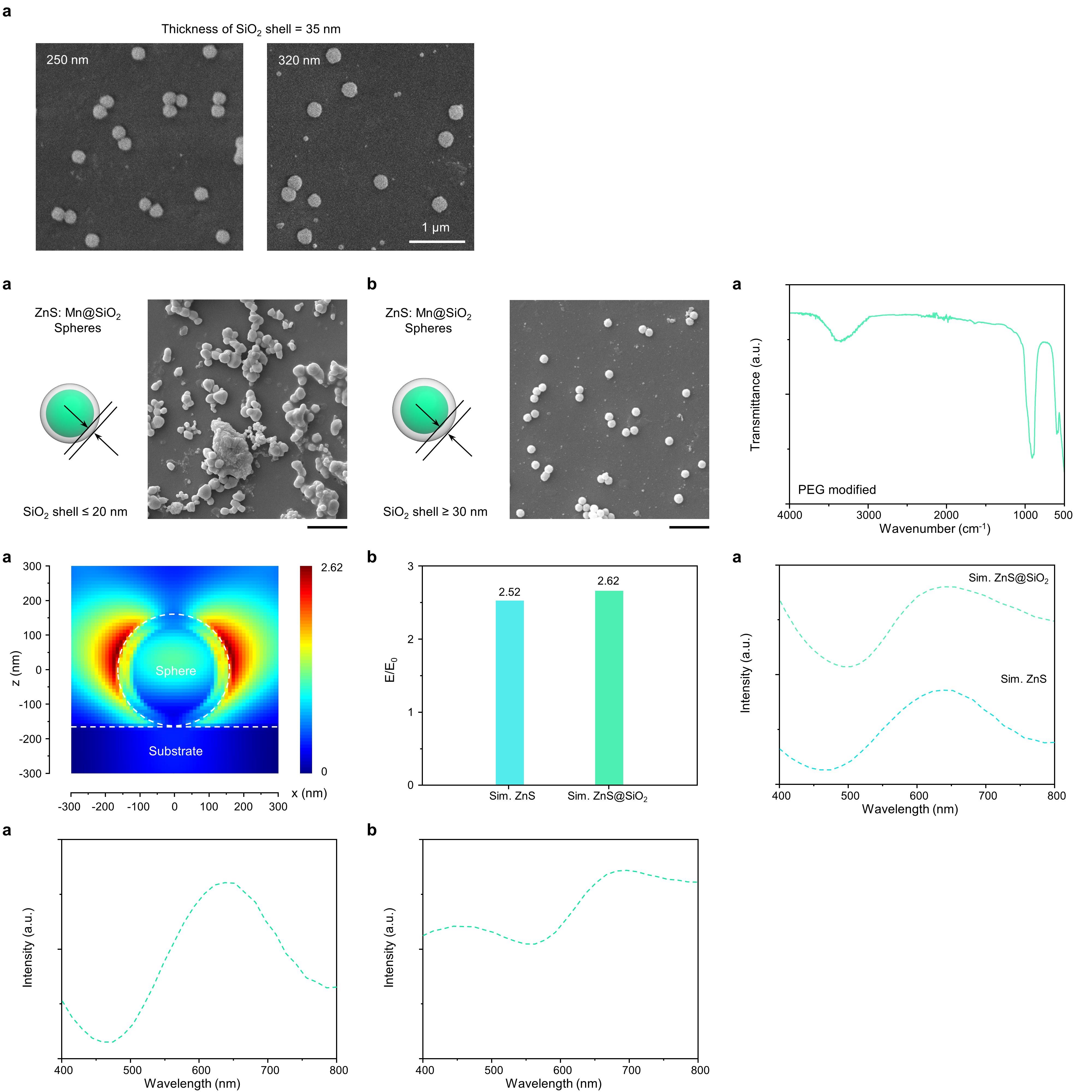


**Figure S10.** a) The simulated electromagnetic field distribution diagram of a single ZnS@SiO_2_ spheres, b) The comparison diagram of electromagnetic intensity.


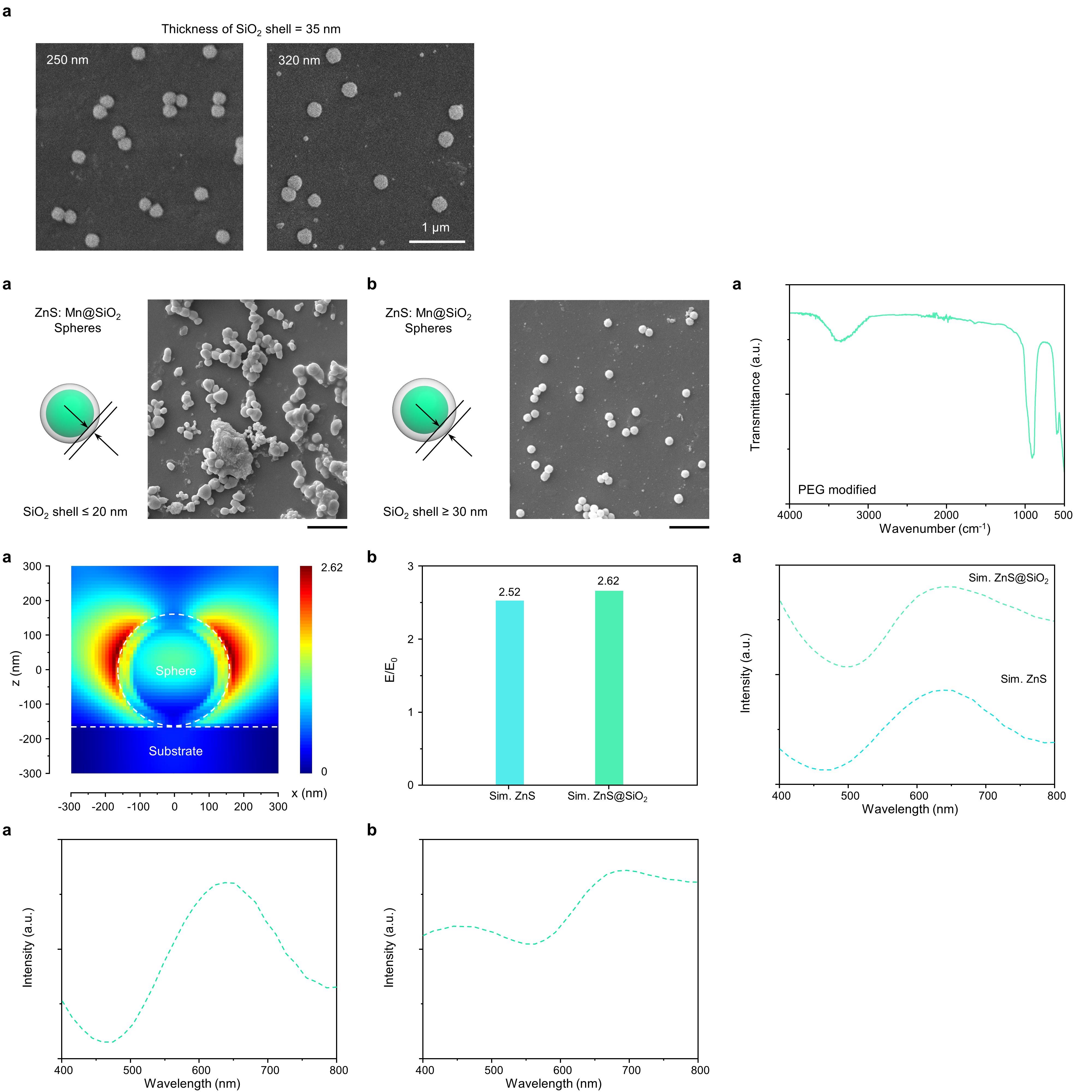


**Figure S11.** The simulated spectra of the single ZnS@SiO_2_ and ZnS sphere.


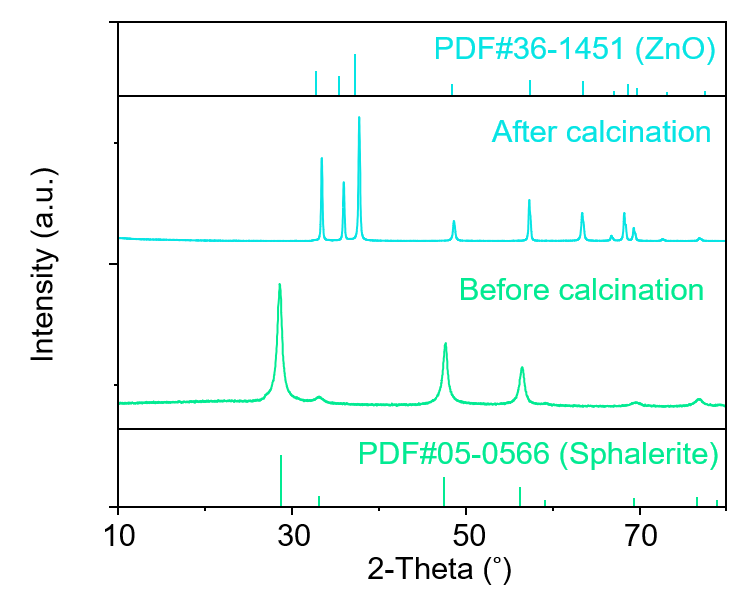


**Figure S12.** XRD spectra of ZnS: X@SiO_2_ spheres before and after 1000 ̊C calcination treatment.


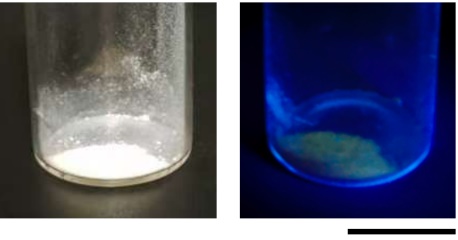


**Figure S13.** Digital photographs of the powder under at oxygen environment. Scale bar: 1 cm.


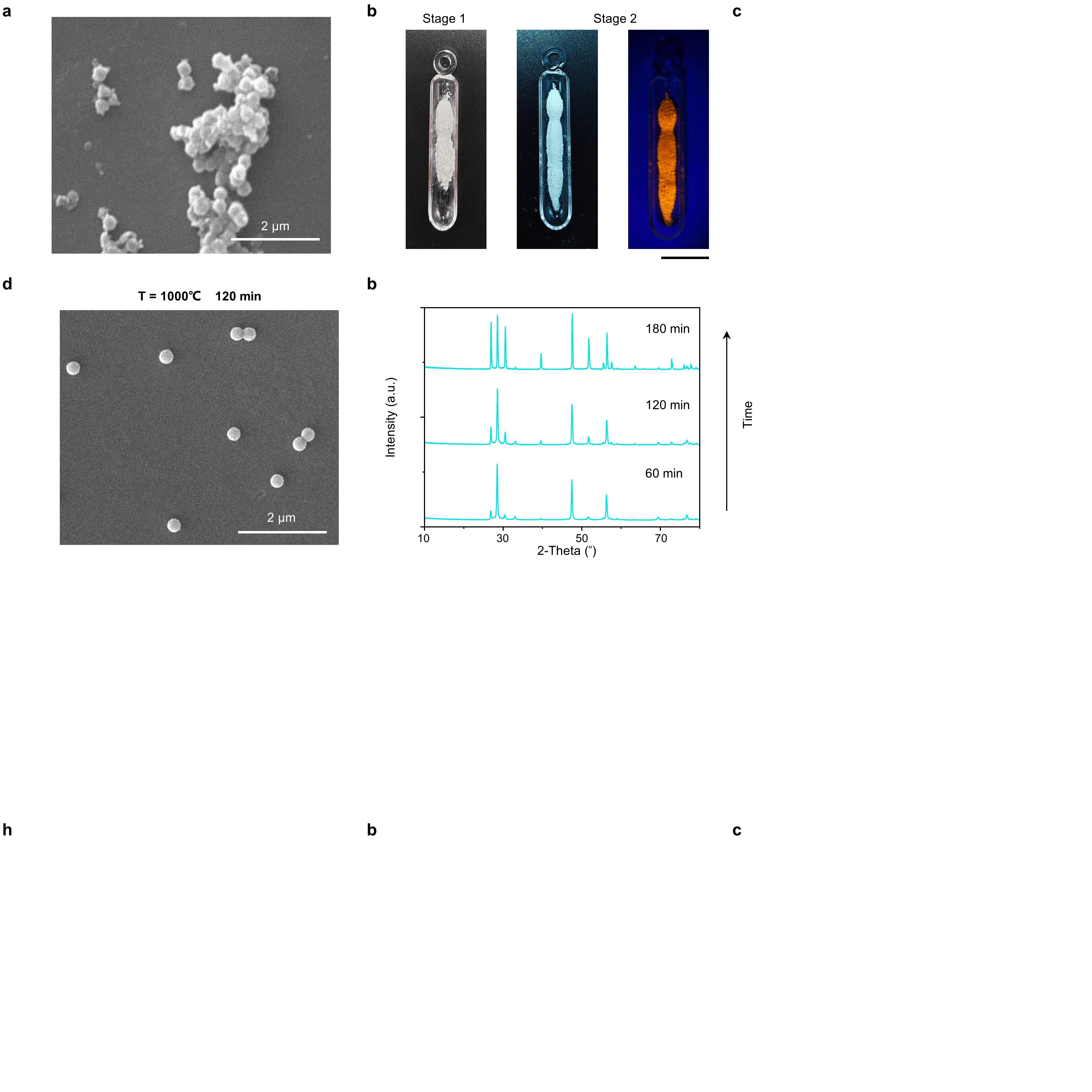


**Figure S14.** SEM image of ZnS: Mn@SiO_2_ spheres after calcination at nitrogen atmosphere.


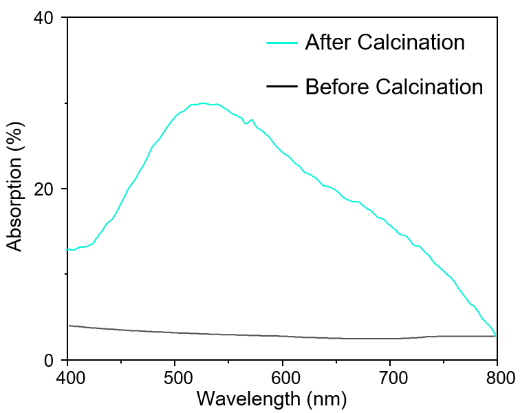


**Figure S15.** Absorption spectra of ZnS: X@SiO_2_ spheres before and after calcination treatment.


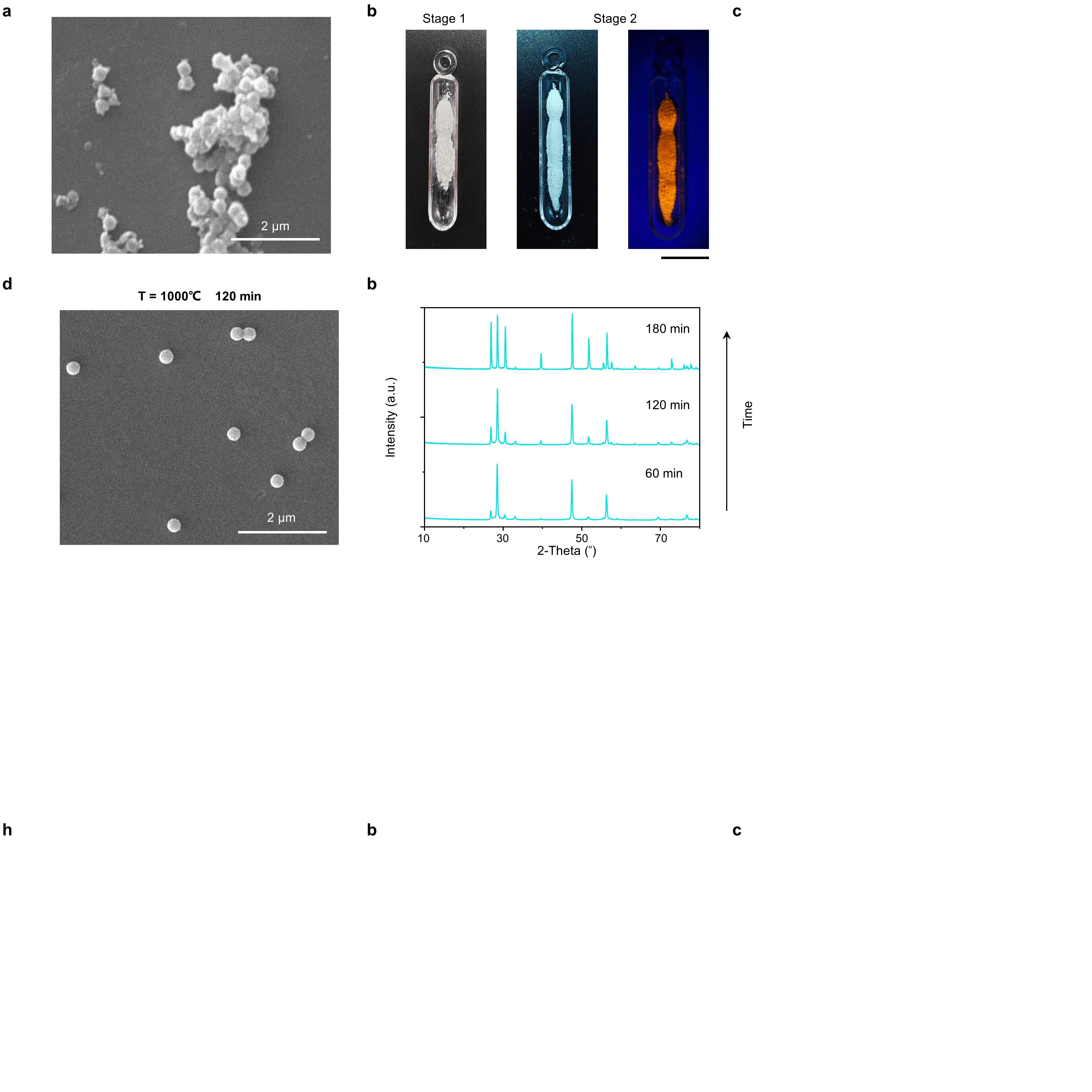


**Figure S16.** Digital photograpghs of ZnS: Mn@SiO_2_ spheres at different stage. Scale bar: 1 cm.


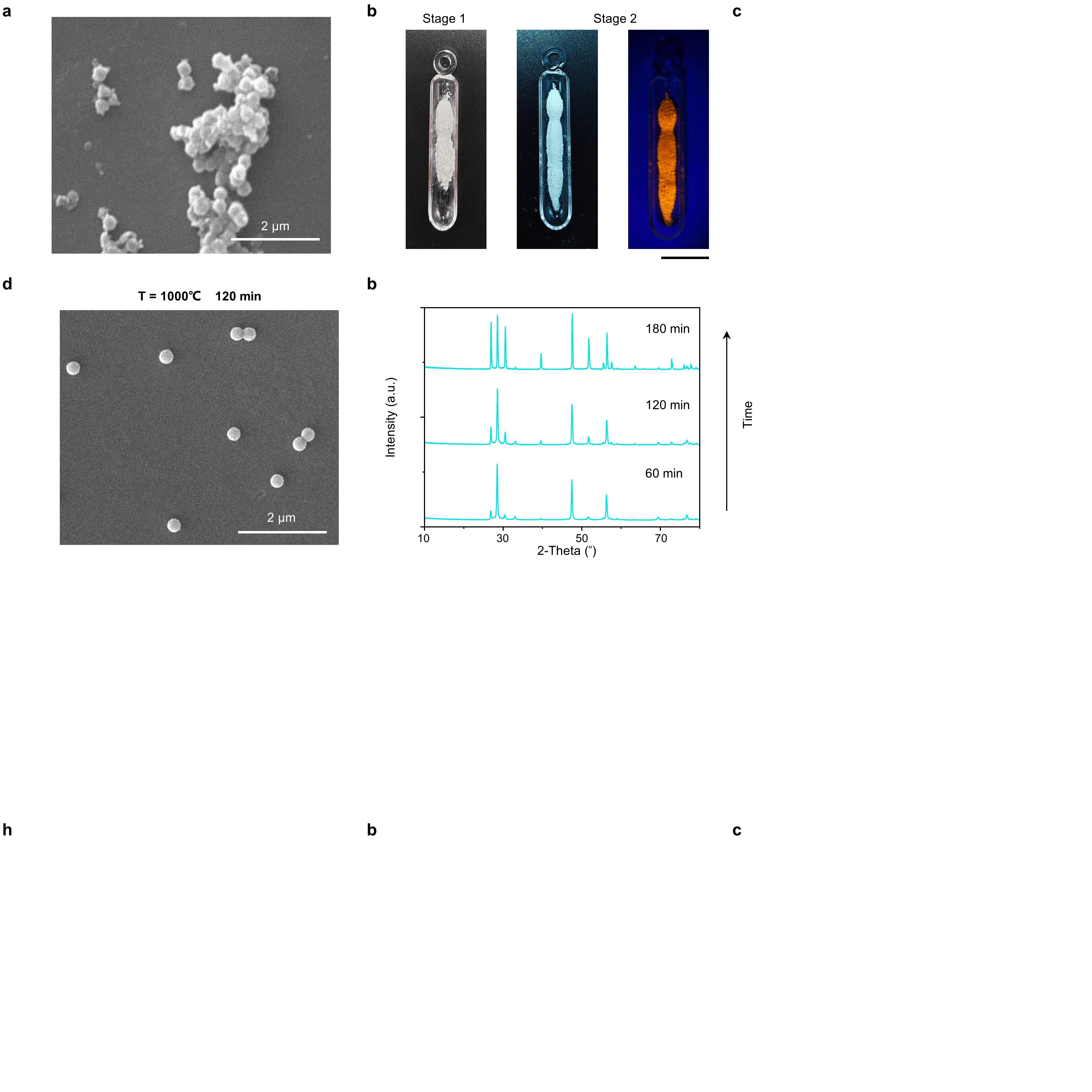


**Figure S17.** XRD spectra of ZnS: Mn@SiO_2_ spheres at different stage.


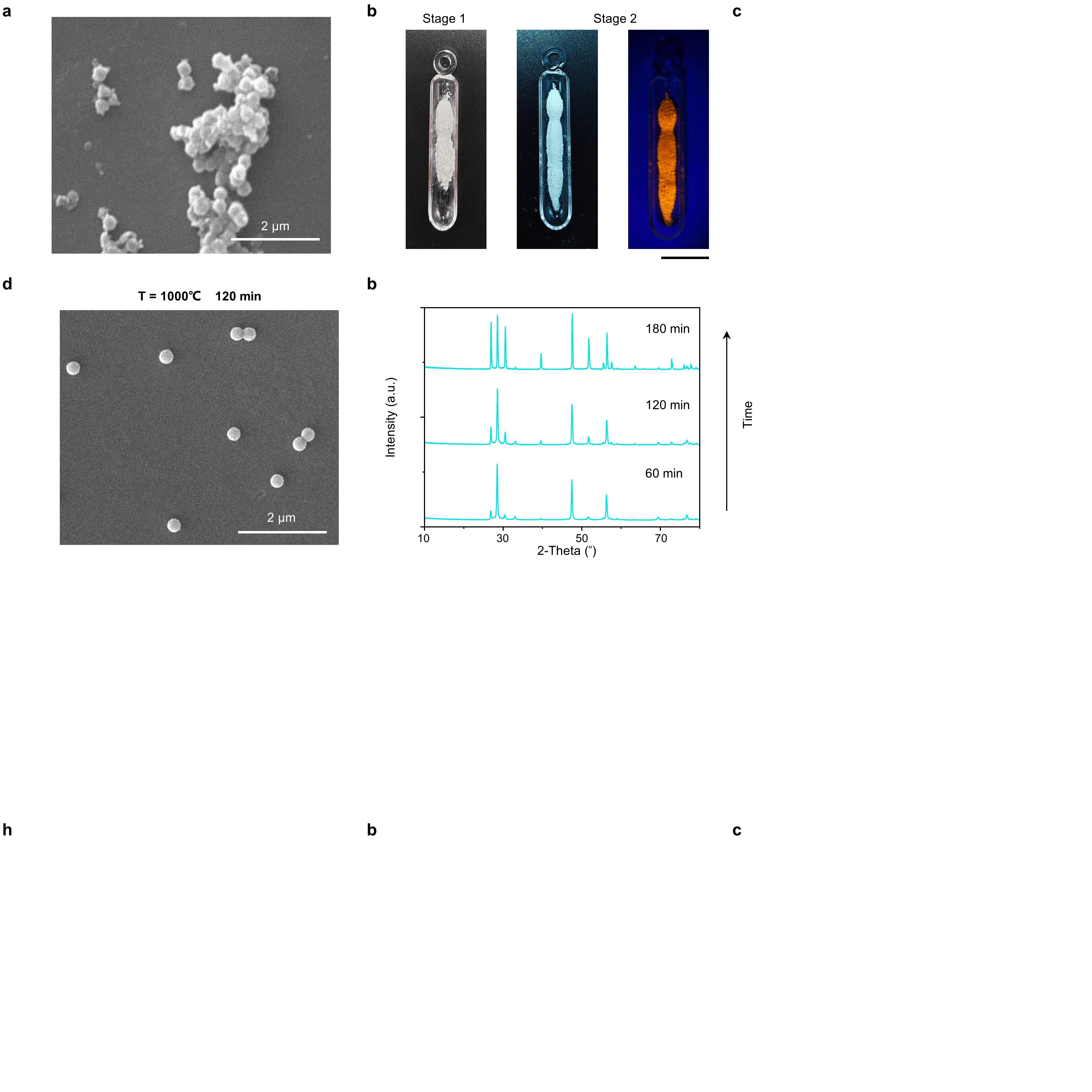


**Figure S18.** SEM image of ZnS: Mn@SiO_2_ spheres fabricated by calcination with an excessive heating rate.


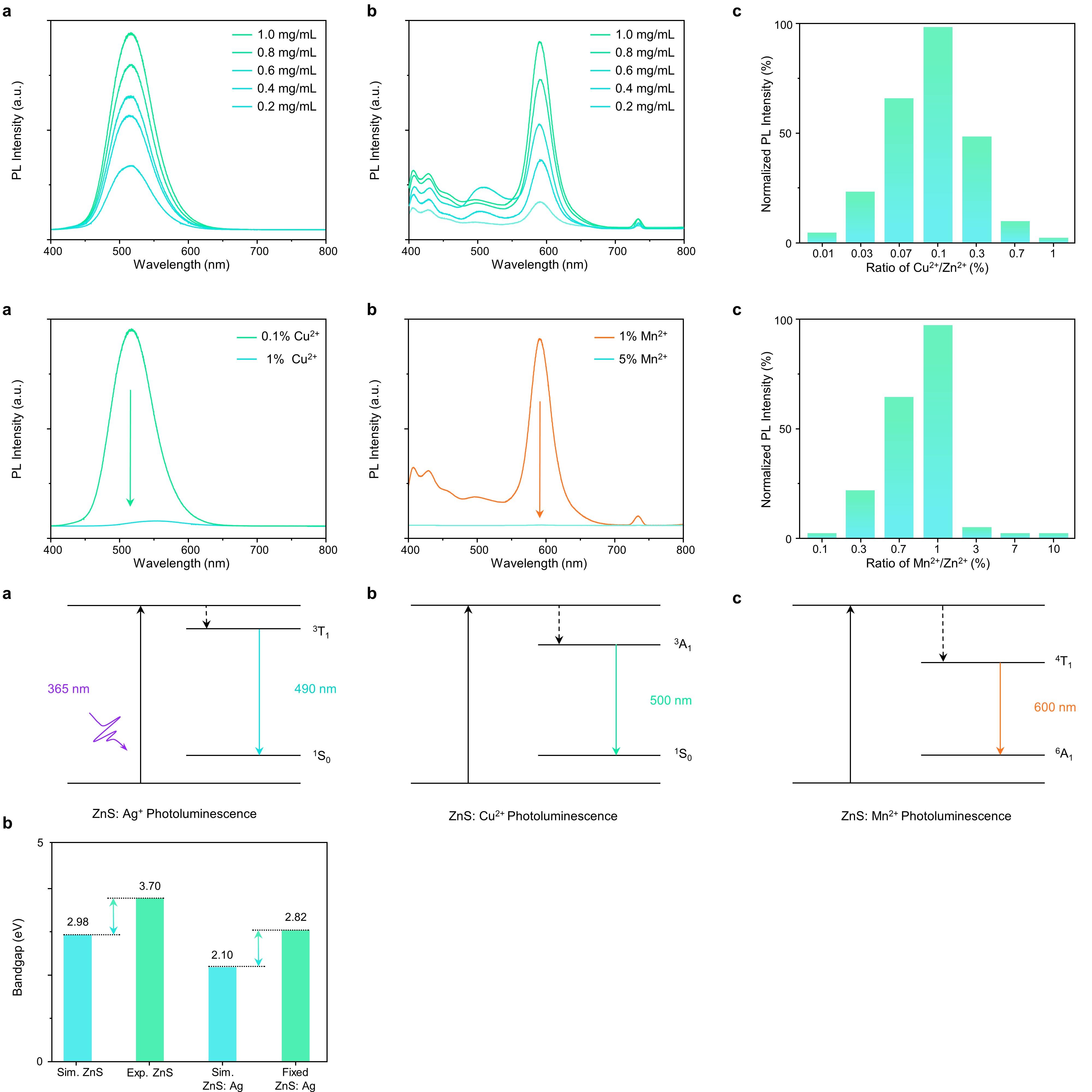


**Figure S19.** The crystal band gap width corrected by the scissors difference operator.


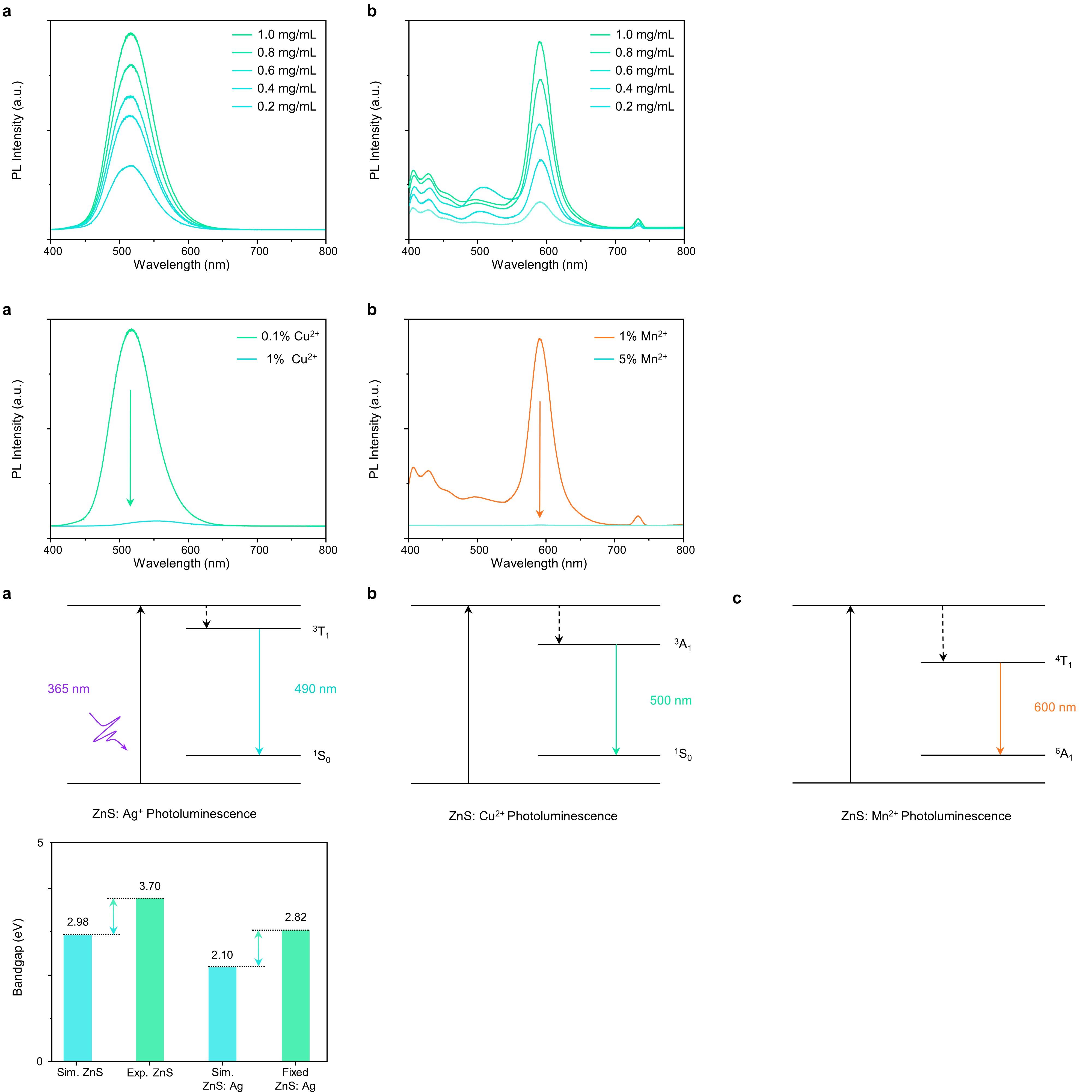


**Figure S20.** The photoluminescence mechanism of ZnS: X@SiO_2_ spheres. a) Ag^+^, b) Cu^2+^, c) Mn^2+^.


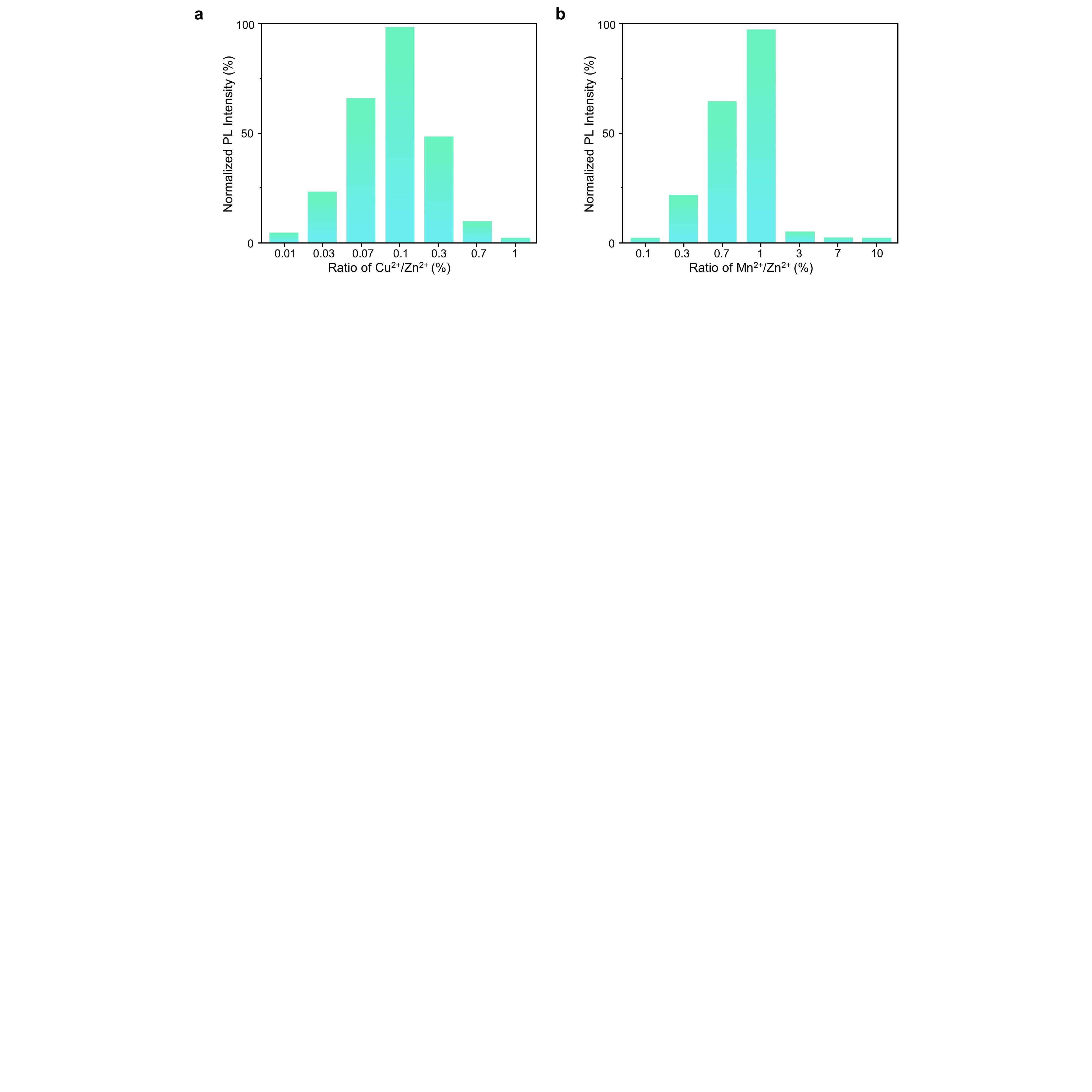


**Figure S21.** a) Normalized PL intensity of wurtzite-type ZnS: Cu^2+^@SiO_2_ spheres with different ratio of Cu^2+^/Zn^2+^(0.01, 0.03, 0.07, 0.1, 0.3, 0.7, and 1%). b) Normalized PL intensity of wurtzite-type ZnS: Mn^2+^@SiO_2_ spheres with different ratio of Mn^2+^/Zn^2+^(0.01, 0.03, 0.07, 0.1, 0.3, 0.7, and 1%).


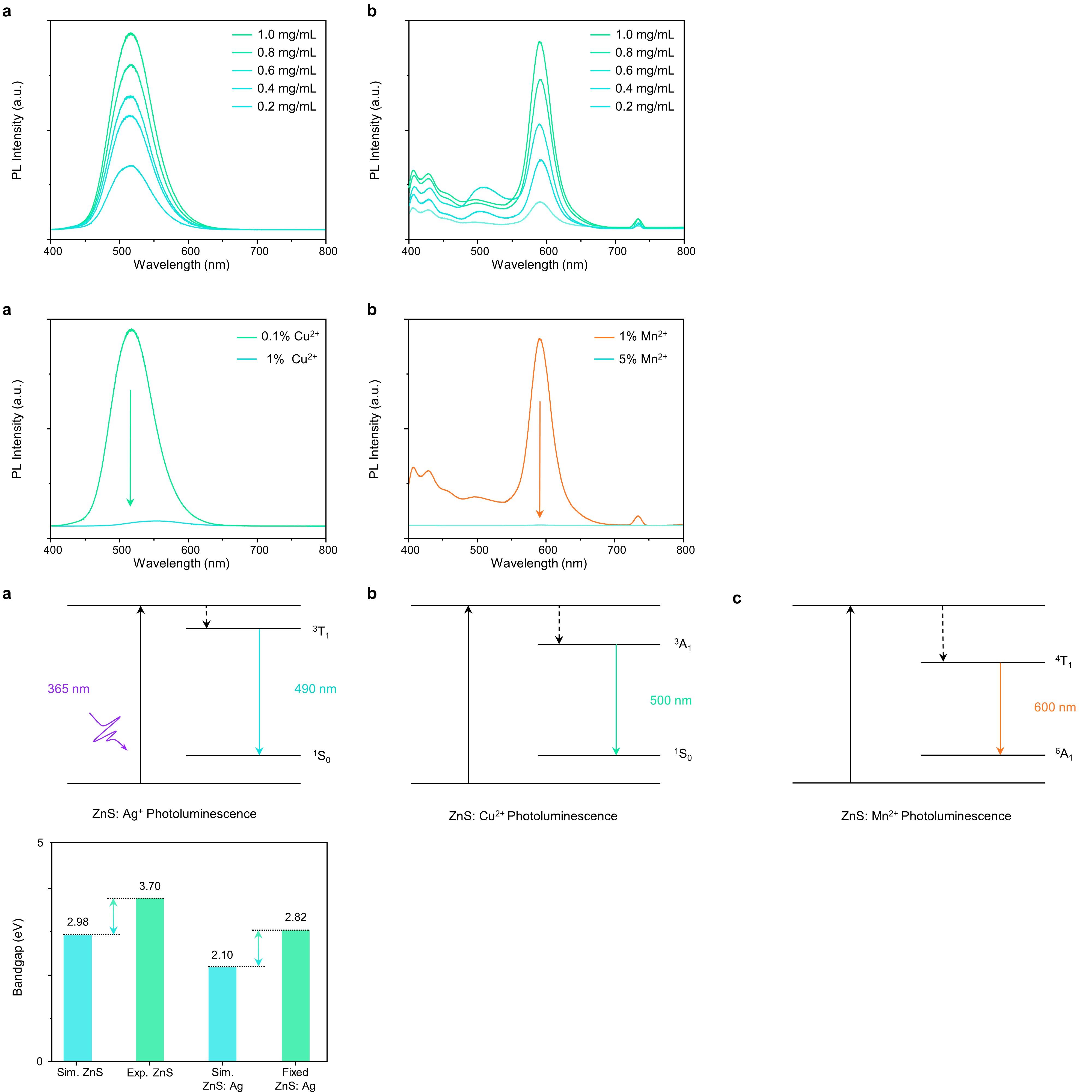


**Figure S22.** a) PL spectra of wurtzite-type ZnS: Cu^2+^(0.1% and 1%)@SiO_2_ spheres. b) PL spectra of wurtzite-type ZnS: Mn^2+^(1% and 5%)@SiO_2_ spheres.


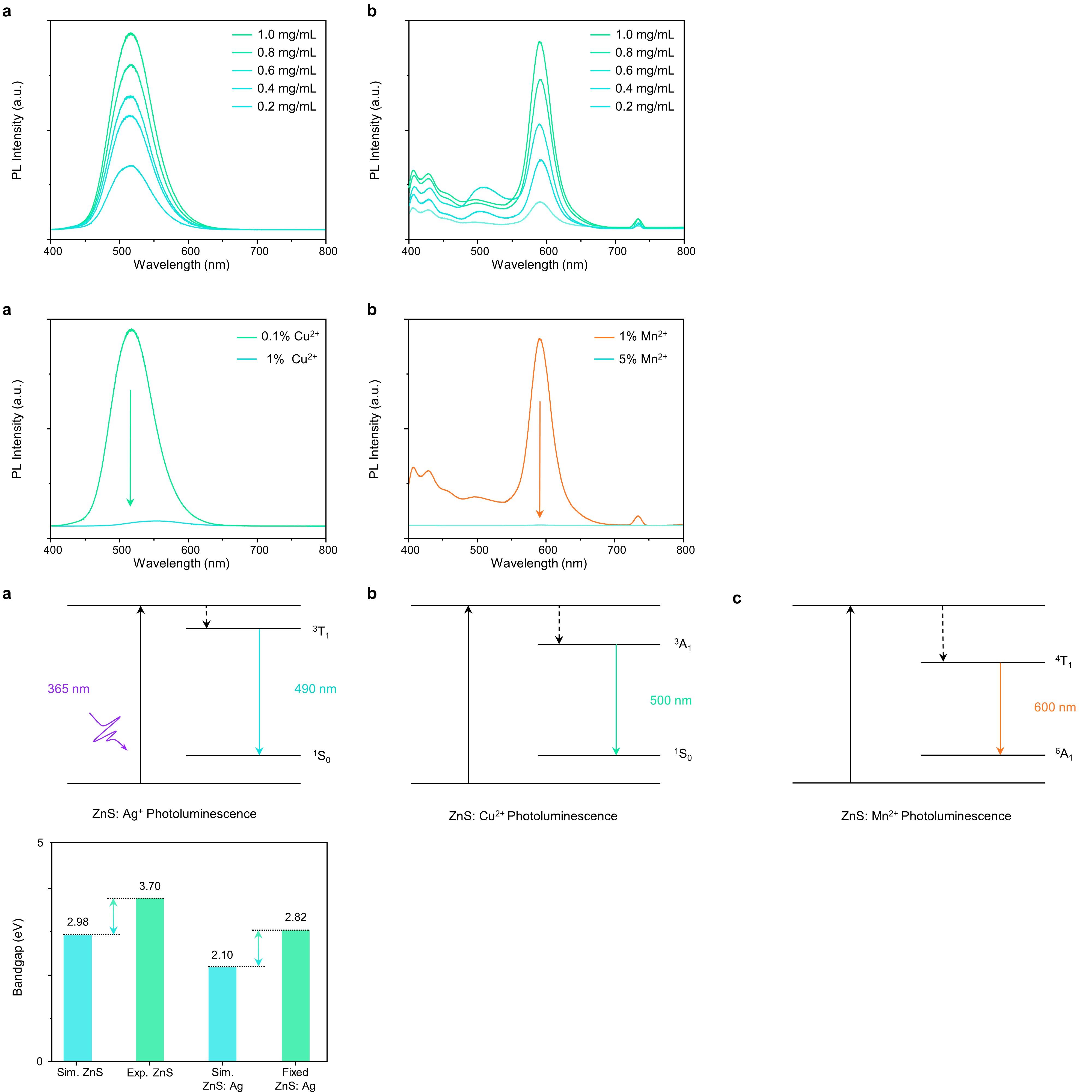


**Figure S23.** a) PL spectra of wurtzite-type ZnS: Cu^2+^(0.1%)@SiO_2_ spheres with different concentration (0.2, 0.4, 0.6, 0.8, 1.0 mg/mL). b) PL spectra of wurtzite-type ZnS: Mn^2+^(1%)@SiO_2_ spheres with different concentration (0.2, 0.4, 0.6, 0.8, 1.0 mg/mL).


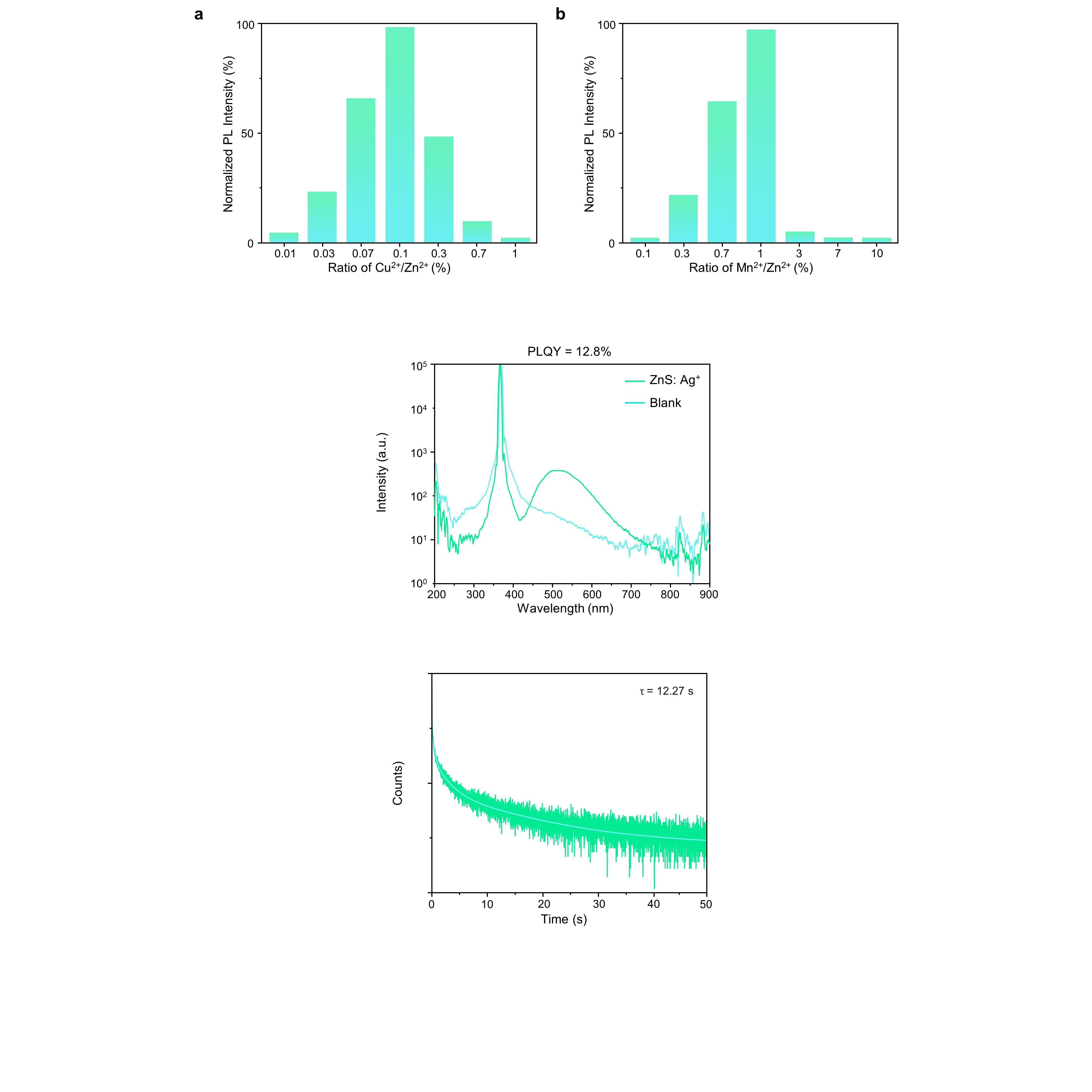


**Figure S24.** PLQY of ZnS: Ag^+^@SiO_2_ spheres.


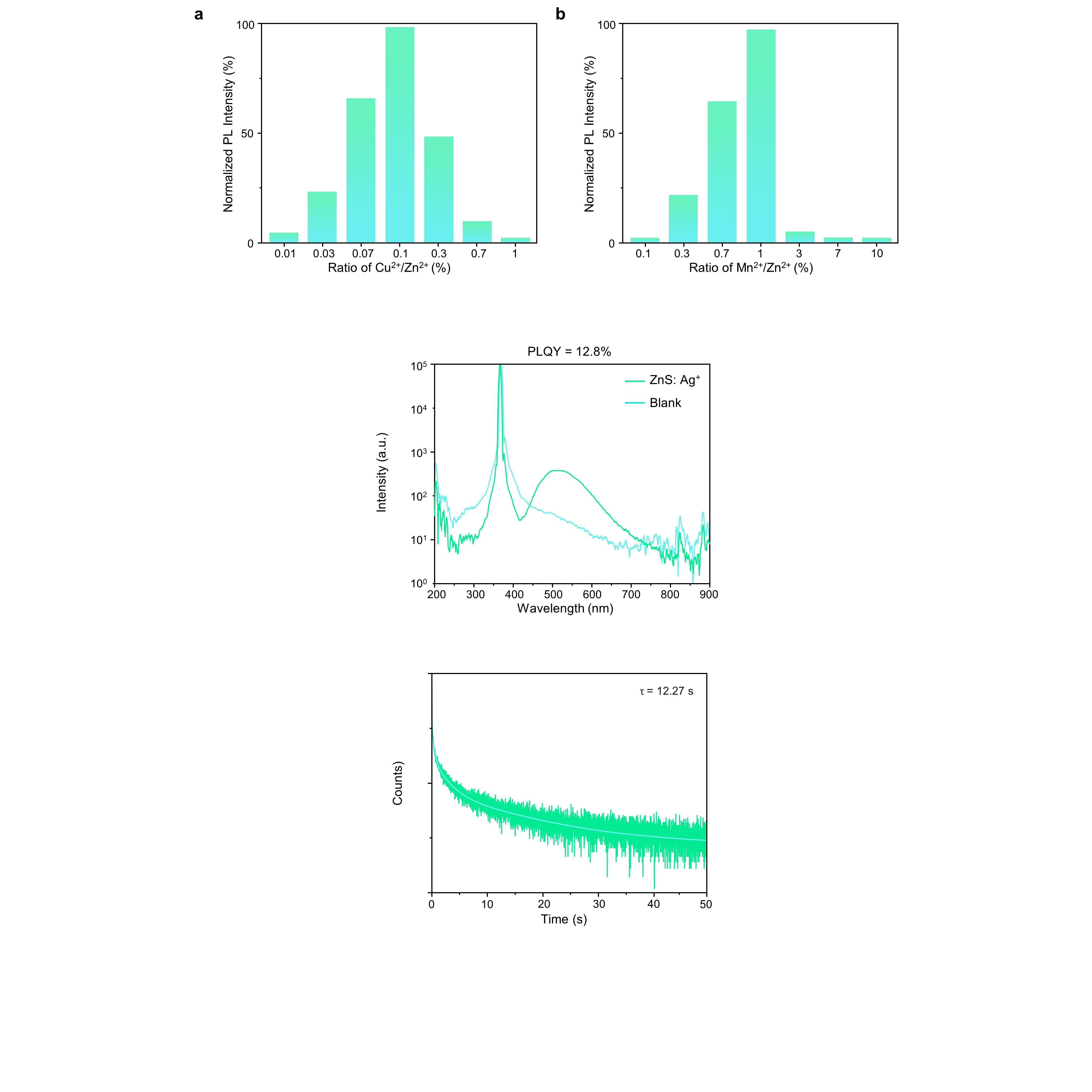


**Figure S25.** Lifetime of the afterglow.


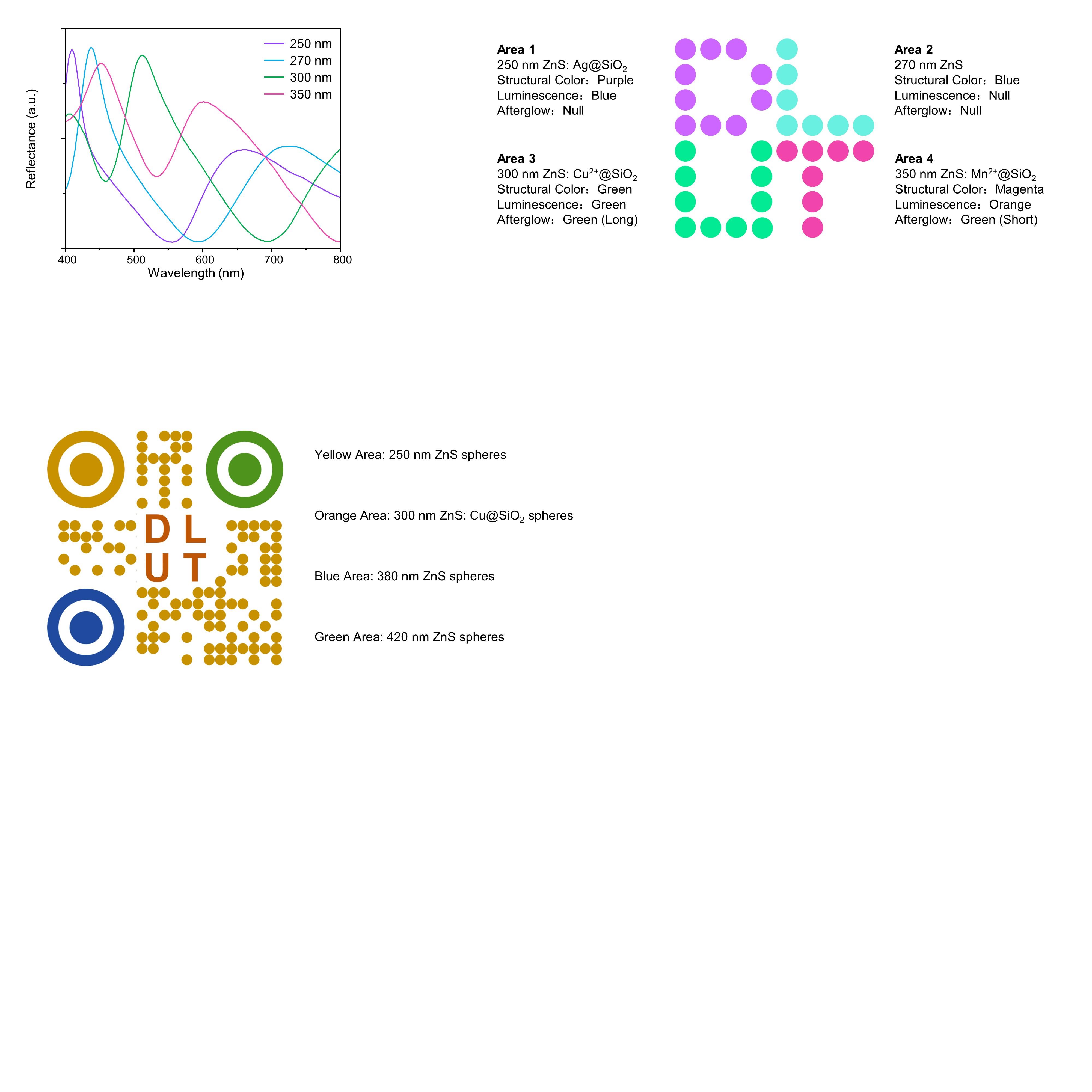


**Figure S26.** Spectra of ZnS spheres with different diameter (250, 270, 300, 350 nm).


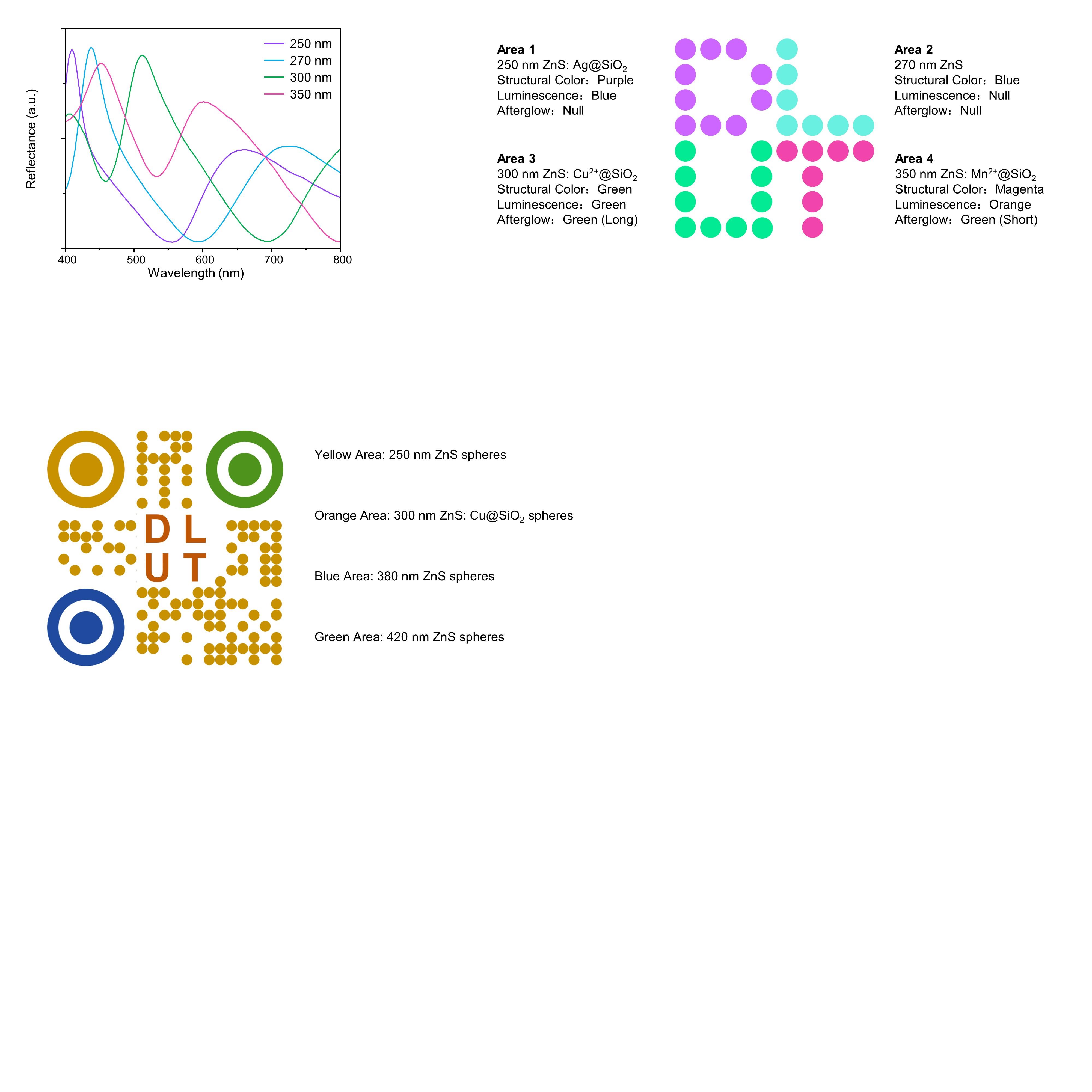


**Figure S27.** Design of the “DLUT“ pattern.


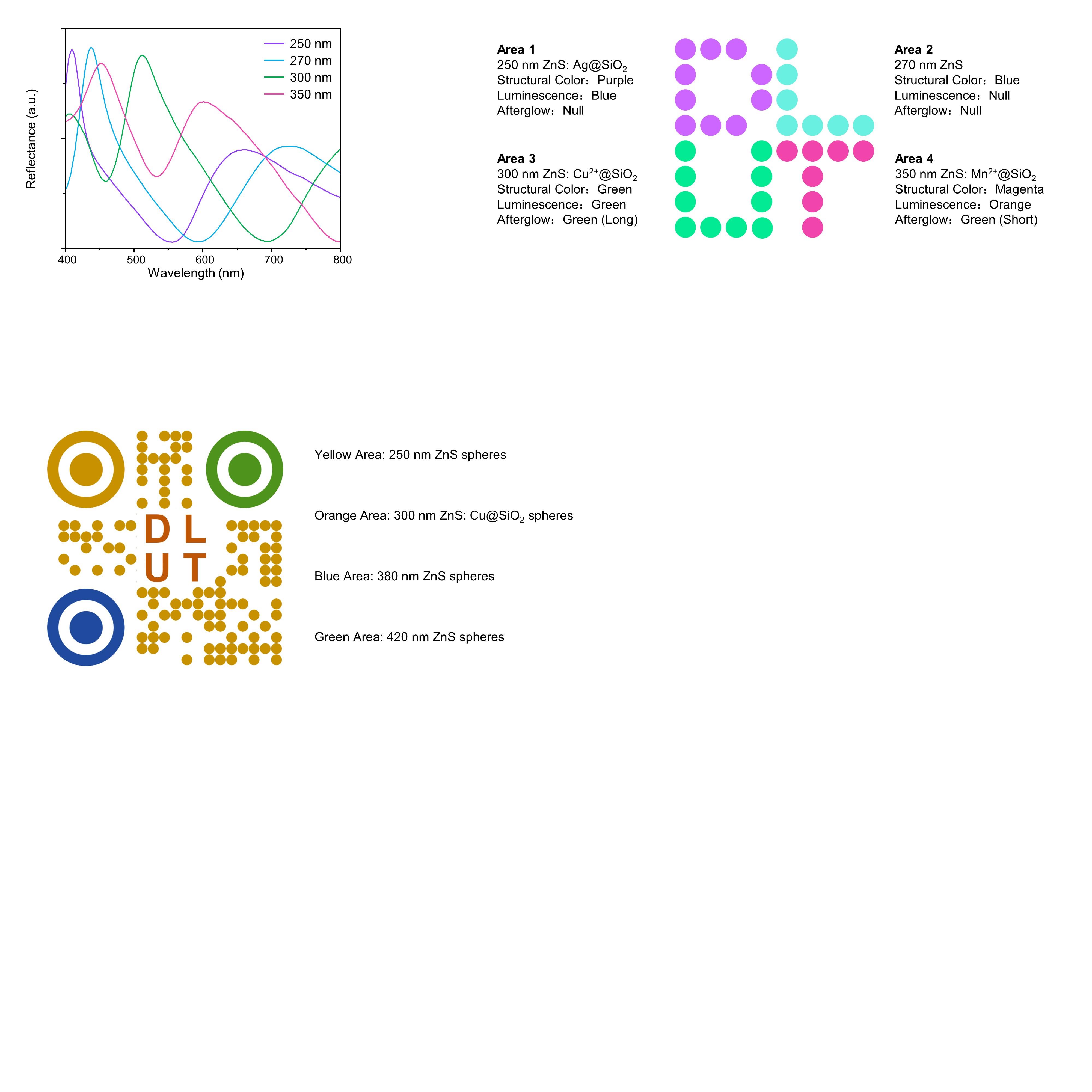


**Figure S28.** Design of the QR-code pattern.
